# Spiramycin (SPR) antibiotics degradation with WO_3_ photocatalyst and ML XGBoost model for process parameters screening

**DOI:** 10.1371/journal.pone.0354585

**Published:** 2026-08-21

**Authors:** Hayat Khan

**Affiliations:** Department of Chemical Engineering, College of Engineering, King Faisal University, Al Asha, Saudi Arabia; Universidad Autónoma de Querétaro: Universidad Autonoma de Queretaro, MEXICO

## Abstract

The intensification of worldwide urbanization and industrial growth has led to a rise in the release of toxic substances into the environment. Antibiotics are widely used as human medicine and animal husbandry, for example annual global consumption (10^6^ kg) of amoxicillin is 11.8, sulfamethoxazole is 2.0, tetracycline is 0.5 and spiramycin is 0.3 etc., therefore, they are often found in surface water and wastewater, thus possess a threat both to human and eco health. Photocatalytic technology can efficiently eliminate highly hazardous, low-concentration, and hard-to-treat contaminant, and tungsten trioxide (WO_3_) serves as a highly promising alternative photocatalyst. Evaluating the effectiveness of WO_3_ in photocatalytic decomposition through traditional techniques is resource intensive and complex. Consequently, in this study experimental results are applied for modeling and optimizing the operational parameters on photocatalytic decomposition of spiramycin, an emerging antibiotic contaminant using WO_3_ as photocatalyst. Three experimental parameters (photoreactor solution pH, WO_3_ concentration and spiramycin (SPR) initial concentration) were chosen to gather preliminary information. The influencing interaction effects and optimal parameters values were determined by using a machine learning technique of XGBoost employing a data set of 45 dataset points obtained under 3 parametric effects. The model achieved an R^2^ value of 0.983 and 0.992 on test and training data, respectively. SHAP (SHapley Additive exPlanations) analysis was utilized to elucidate the prediction outcomes, uncovering the importance and influence patterns of the input parameters. The degradation rate of SPR approached ~87.6% under optimal conditions of pH value of 5.0, WO_3_ concentration of 0.125g/l and pollutant concentration of 0.15 g/l, respectively. In addition, the applied ML method revealed that the most influencing parameter is the reactor solution pH value followed by pollutant concentration on the decomposition of SPR antibiotic. Moreover, we conducted experiments to investigate the role of oxidizing species. Our findings indicate that primarily hydroxyl radicals (OH^•^) followed by photoinduced holes (h^+^s) and hydrogen peroxide (H_2_O_2_), play significant roles in the breakdown of model contaminant. We also studied the reaction kinetics and proposed the photocatalytic mechanism based on the obtained results. As a study outcome, the XGBoost model exhibits great accuracy and strength, suggesting that machine learning holds considerable practical promise and worth in forecasting the degradation emerging pollutants via photocatalysts.

## 1. Introduction

Water pollution has become a major environmental challenge associated with modern industrialization. The issue of unembellished water contamination involves pharmaceutical compounds such as psychiatric drugs, analgesics, anti-viral, hormones, antibiotics and anticancer drugs, which possess a serious health risk in the modern world for both humans and aquatic life in water, in addition to damage to land fertility. It is important to note that antibiotics are commonly used in hospitals to treat bacterial illnesses, therefore, it is reasonable to expect high concentrations of these medications in hospital effluent [1]. The primary sources antibiotic contamination in soil and water bodies include antibiotic production businesses, inappropriate antibiotic waste management, the limited removal efficiency of conventional wastewater treatment facilities (WWTPs), and agricultural and veterinary operations [2]. It was reported elsewhere [3] that from 2016–2023 the estimated amount of consumed antibiotics increased 16.3% from 29.5 to 34.3 billion defined daily doses (DDDs). This represents a 10.6% rise in the daily consumption rate, from 13.7 to 15.2 DDDs per 1,000 inhabitants. In addition, it was predicted that global antibiotic consumption will rise by 52.3% from an estimated 49.3 billion DDDs in 2023 to 75.1 billion DDDs by 2030 if nothing is done in quickly developing countries, such as investing in better infrastructure, specifically water and sanitation, and better access to vaccination. Many approaches, including membrane bioreactors (MBRs) systems, evaporation, artificial wetlands, conventional activated sludge, anaerobic baffled reactors, coagulation, adsorption, advanced oxidation technology, the membrane process, and combinations of these approaches, have been put forth to eliminate antibiotics and other related substances. Recently, among these techniques adsorption and advanced oxidation processes (AOPs) have attracted a lot of interest from researchers delivering a significant amount of research work. AOPs are dependent on the in-situ generation of highly reactive hydroxyl radicals (OH^•^) with the aid of one or more primary oxidants (such as O_2_, H_2_O_2_ and O_3_), energy sources (such as UV, solar, and visible light), and/or catalysts (such as TiO_2_, ZnO and WO_3_) [4,5]. Refractory water contaminants react with OH^•^ resulting in their breakdown or mineralization into inorganic ions, CO_2_, and H_2_O. To date, a number of AOPs have been used to remove residual pharmaceutical compounds, including hybrid AOPs, Fenton and photo-Fenton, ozonation, electrochemical, ultrasonic or sonolysis, and heterogenous photocatalysis [6]. In particular, heterogeneous photocatalysis has demonstrated significant promise as a flexible, affordable, sustainable, efficient and eco-friendly AOP treatment method for the elimination of persistent organic compounds in wastewater. In case of pharmaceutical compounds, heterogenous photocatalysis has been applied to remove β-blockers, steroid estrogen, antibiotics, and anticonvulsants [7]. During photocatalysis, conduction band (CB) electrons (e^−^s) and valence band (VB) holes (h^+^s) are produced upon catalyst activation via suitable light energy. Subsequently, reactive species such as superoxide anions O_2_^•-^, H_2_O_2_ and OH^•^ radicals are formed, which drive the decomposition of pollutants [8].

Among all functional nanomaterials, tungsten oxide (WO_3_) is a projecting semiconductor oxide, a visible light active photocatalyst with a forbidden bandgap of roughly 2.6 to 2.8 eV. With a VB potential of about 2.7–3.44 eV, the holes generated in the VB exhibit a robust oxidation capability akin to that of the widely utilized TiO_2_ photocatalyst. In addition, it is fairly plentiful in nature, medium hole diffusion length (~150 nm), good electron mobility (~12 cm^2^/V s), positive valence band edge (3.0 V vs. NHE) for water oxidation [9], is non-toxic, demonstrates good corrosion resistance in acid electrolyte, and exhibits physical and chemical stability.

Several studies have investigated the removal efficiency of WO_3_ photocatalysts for the decomposition of pharmaceutical antibiotics. Beheshti et al. [10] employed commercial WO_3_ for the photocatalytic degradation of sulfamethoxazole antibiotic, under optimum conditions of pH value of 3, catalyst dosage of 750 mg/l, SMX concentration of 50 mg/l and halogen lamp contact time of 90 min results in 93.96% degradation. Nguyen et al. [11] used commercial WO_3_ for the decomposition of amoxicillin (AMO) antibiotics by using simulated solar irradiation of 300-W xenon lamp. Under optimal conditions, an initial AMO concentration of 1.0 μM, a pH of 4, irradiation time of 180 min and a catalyst dosage of 0.104 g/l results in almost complete removal of 99.99%. Yang et al. [12] used electrochromic WO_3_ nanocluster electrocatalyst synthesized by anodization for the complete dehalogenation of florfenicol antibiotic. The ideal WO_3_/W anodized at 25 V yielded the greatest electrocatalytic dehalogenation performance, 99.7% in 120 min. Moreover, after 20 hours of operation, the self-supporting WO_3_ nanocluster electrocatalyst exhibits no loss of, indicating strong potential for the development of a workable, affordable, and durable electrochemical method for antibiotic dehalogenation. Similarly, Quyen et al. [13] examine the capability and effectiveness of Cu-WO_3_ for the increased photocatalytic degradation of tetracycline (TC) antibiotic in wastewater. Experimental results showed that 2.5 wt.% doped Cu loaded on WO_3_ exhibit the enhanced TC decomposition efficiency of 96.8% within 120 min under visible light. The enhanced activity was attributed to increased photoinduced charge separation and visible light response due to the narrowed band gap energy of 2.6 eV. Zhu et al. [14] employed pure WO_3_ and Ag loaded WO_3_ nanoplates for effective photocatalytic removal of sulfanilamide (SAM) antibiotic under visible irradiation. The findings demonstrate that WO_3_/Ag composites outperformed pure WO_3_, with the maximum removal rate of 96.2% in 5 hr. The enhanced e^-^/h^+^ pair separation rate, where Ag nanoparticles function as an efficient electron trapper during the photocatalytic process, is responsible for the better performance in terms of SAM decomposition with WO_3_/Ag.

In nutshell, these studies are not limited to, which clearly demonstrate that WO_3_ has the potential to degrade pharmaceutical antibiotics. Therefore, in this study we take on the assignment being first of its kind to apply self-synthesized WO_3_ for the photocatalytic decomposition of an emerging antibiotic, i.e., Spiramycin (SPR). Spiramycin is an antiparasitic and antibiotic agent derived from *Streptomyces ambofaciens and* approved by the British Pharmacopoeia Commission. In European countries, it is usually referred to as Rovamycin. Spiramycin, which belongs to the macrolide antibiotics with a 16-membered lactone ring, is commonly utilized in both human and veterinary medicine [15] to treat infection caused by gram +ve and gram -ve bacteria such as Streptococci, Pneumococci, Leginella spp., Mycoplasma and Toxoplasma gonidia. Due to its usage SPR belongs to a family of compounds known as “emerging contaminants,” which have the potential to enter the environment and cause harm across all tiers of the biological hierarchy. Thus, developing effective and long-lasting methods for reducing and degrading antibiotic remnants in water bodies is crucial. Moreover, enough literature is available illustrating the application of heterogenous photocatalysis for the degradation of recalcitrant contaminants primarily by standard optimization that looks at one parameter while keeping the rest constant [10,13,16]. It is a time-consuming process that ignores the combined effects of several parameters on photocatalytic degradation. Multiple influencing parameters in photocatalytic reactions can be simultaneously optimized using tree-based machine learning models, for instance, XGBoost, Random Forest (RFs), artificial neural networks (ANNs) etc., allowing affordable, practical, inclusive optimization to reduce the cost and study time.

As a branch of artificial intelligence (AI), machine learning (ML) possesses a unique advantage in addressing intricate and non-linear problems by identifying functional patterns and insights derived from input data [17]. Recently, ML has emerged as a powerful tool for accelerating the discovery and optimization of photocatalysts [18]. ML algorithms can forecast material properties, determine ideal reaction conditions, and minimize the necessity for extensive experimentation by analyzing large datasets [19]. Jaison et al. [20] provided a summary of the incorporation of ML in photocatalysis, emphasizing its contributions to enhancing absorption of light, separation of charge, and design of photoreactors. Ge et al. [21] endowed an insight into advancements in leveraging ML to comprehend the intricate connections between photocatalyst structure and photoactivity and to pinpoint the factors governing activity. Zhang et al. [8] investigated the effect of operational parameters on the decomposition of chloramphenicol employing TiO_2_ nanoparticles followed by a quadratic model development to optimize the process parameters of chloramphenicol decomposition using RSM on the basis of data obtained from conducting the initial experiments. Moreover, Kazim et al. [22] examined the applications of artificial intelligence models to predict and decomposition rates in photocatalytic activities. Ali et al. [23] conducted a review of the recent developments in ML-guided predication of CO_2_ photocatalytic reduction. Even though the aforementioned literature offers various perspectives on ML (RSM, ANNs) applications in photocatalytic technology, however, there remains substantial scope to explore the applications of ML for examining the effect of different parameters on the photocatalysis phenomena. Therefore, to effectively address the severe issues of antibiotic contamination, it is necessary to modify the traditional single-factor approaches and adopt more comprehensive methodologies that can better guide the actual process. To the best of our knowledge, no study has been published that focuses on optimizing operational parameters for the photocatalytic degradation of spiramycin (SPR) antibiotics using WO_3_ as a photocatalyst with an XGBoost ML model. The current study examined the influence of three process factors (pH, WO_3_ concentration, and SPR initial concentration) on SPR decomposition rate (variation in concentration) in a batch reactor under different experimental conditions. To begin with, the primary data was acquired to identify the optimal conditions for SPR decomposition, subsequently a tree-based machine learning (ML) model namely XGBoost model was developed. The effects of influential factors were assessed on the basis of SHAP analysis. The developed model demonstrated better performance even compared with Random Forest (RF), offering a new and efficient approach for predicting photocatalytic activity on the basis of initial experiments.

To further strengthen the study, we have analyzed kinetic data by adopting the Langmuir–Hinshelwood (L–H) kinetic model and performed radical scavenging experiments (ROS) to examine the radicals (HO^•^, O_2_^-•^ etc.) or holes (h^+^s) liable for the model pollutant decomposition. In addition, total organic carbon (TOC) analysis and photocatalyst recyclability tests were performed and finally mechanistic study was also devised. This study provides a novel approach to addressing SPR antibiotic contamination, while also offering valuable insights for predicting and optimizing the SPR degradation process.

## 2. Experiments

### 2.1. Materials

**Fig 1:**
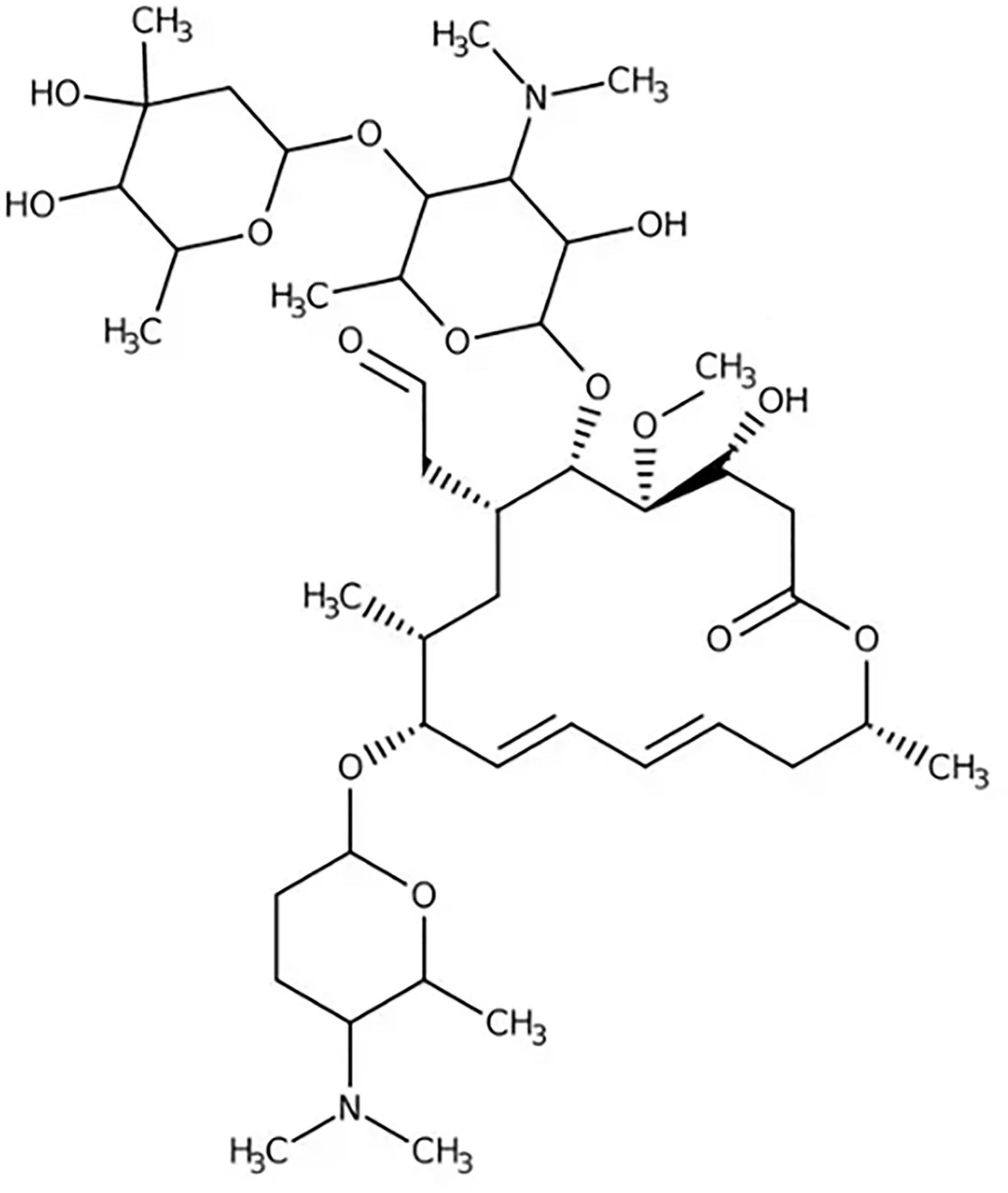
Spiramycin structure (CAS:8025-81-8).

Spiramycin (C_43_H_74_N_2_O_14_, MW-843.065 g/mol, purity ≥ 90%, water solubility-0.196 mg/ml) was obtained from Fisher Scientific, Canada. Its structure is shown in Fig 1.

The photocatalyst nano WO_3_ (light yellow color, crystallite size 24.0 nm, band gap 2.62 eV, needle like morphology, BET surface area 99 m^2^/g, pore diameter 1.7 nm, monoclinic crystalline structure (ICDD ref. card no. 43–1035) and bandgap value of 2.62 eV) was synthesized by crash precipitation technique followed by spray drying and calcined at 600 °C. The detailed synthesis procedure (as also described in the following section) and characterization details are reported elsewhere [24]. All other chemicals (HNO_3_, NaOH) were of analytical reagent grade and were obtained from Sigma Aldrich and Fisher Scientific, Canada. Every chemical was used just as it was delivered, requiring no additional purification. Throughout this research work, laboratory deionized (DI) water was utilized.

### 2.2. Photocatalyst synthesis

A crash precipitation method was employed to synthesis WO_3_ nanoparticles. Ammonium paratungstate (NH_4_)10H_2_(W_2_O_7_)_6_), hydrochloric acid (HCl), and water with a molar ratio of 0.005:200:500. After dissolving ammonium paratungstate in HCl while stirring, the mixture was quickly added to water, causing a yellow-white precipitate of WO_3_ to precipitate. After removing the excess water from the precipitate, we repeatedly cleaned it with deionized water (4 × 100 ml). To create the WO_3_ suspension, we finally added 20 ml of deionized water to the precipitate while continuously stirring. In the second step, we followed the same procedure stated in our previous paper [25], as well as the same equipment (Yamato GB-22 dryer) to produce the spray dried WO_3_ powders. Finally, the crystalline WO_3_ material was produced by annealing the spray dried powders for two hours at 600 °C in a programable muffle furnace using a heating rate of 3 °C/min. Moreover, the above method is facile and reproduceable, having an uncertainty of < 1 nm in the photocatalyst crystallite size.

### 2.3. Photocatalytic experiments

Room temperature photocatalytic activity of WO_3_ was evaluated by decomposition of spiramycin (SPR) using an acrylic cylindrical batch reactor with a central vertically mounted UV lamp (Germicidal UVC lamp, Atlantic Ultraviolet Corp. GPH212T5L/4, lamp power - 10W, length – 0.212 m, λ_max_ output at 254 nm, light intensity of approx. 26 μW/cm^2^ measured at a distance of 1 m, and maximum incident photon flux per unit volume at the batch reactor mid-section was approximately 1.3 × 10^−3^ Einstein/min l [26]) protected in a quartz sleeve. Before each experiment, photocatalyst (0.15 g/l) and model pollutant SPR (0.1 g/l) were suspended in DI water in different flakes and sonicated for 30 min to decrease particle agglomeration. Both SPR solution and WO_3_ photocatalyst suspension were added to the reactor followed by adding rest of DI water to make the final volume to 1.5 L. The reactor suspension was magnetically stirred in dark for 1 hr to achieve the adsorption equilibrium followed by switching on the UV light for another 1 hr photocatalytic test. During the experiment, filtered air from an external source was bubbled continuously. Samples (2 ml) were taken from the reactor at specific time internal of 10 min and filtered (Millipore filters, porosity 0.22 μm) to separate the particles of the photocatalyst. The photocatalytic degradation of SPR was analyzed on a UV-Vis spectrophotometer by measuring the highest peak absorbance at 232 nm using the calibration curve. Moreover, in all activity tests the reactor pH was monitored by using a Fisher AccumetTMAB15 pH meter equipped with a glass pH electrode. The influence of (i) pH in the range 3−11 (adjusted with 0.5 M NaOH and 0.25 M HNO_3_), (ii) pollutant concentration in the range 0.1–0.2 g/l and (iii) WO_3_ photocatalyst amount in the range 0.05–0.15 g/l were studied. Every photodecomposition test was carried out three times. Isopropyl alcohol (IPA, 0.25 mM), enzyme catalase (22 mg/l), benzoquinone (BQ) and ammonium oxalate (AO, 0.1 g/l) were opted as scavengers in order to examine the production of various reactive oxidizing species (ROS) such as hydroxyl radicals (OH^•^), H_2_O_2_, O_2_^•-^ and h^+^, respectively, throughout the photocatalytic process.

## 3. Machine learning modelling

This section presents data preprocessing of the employed dataset, an overview of the XGBoost model, and explains the model development procedure.

### 3.1. Data development and Pearson’s correlation examination

**Fig 2:**
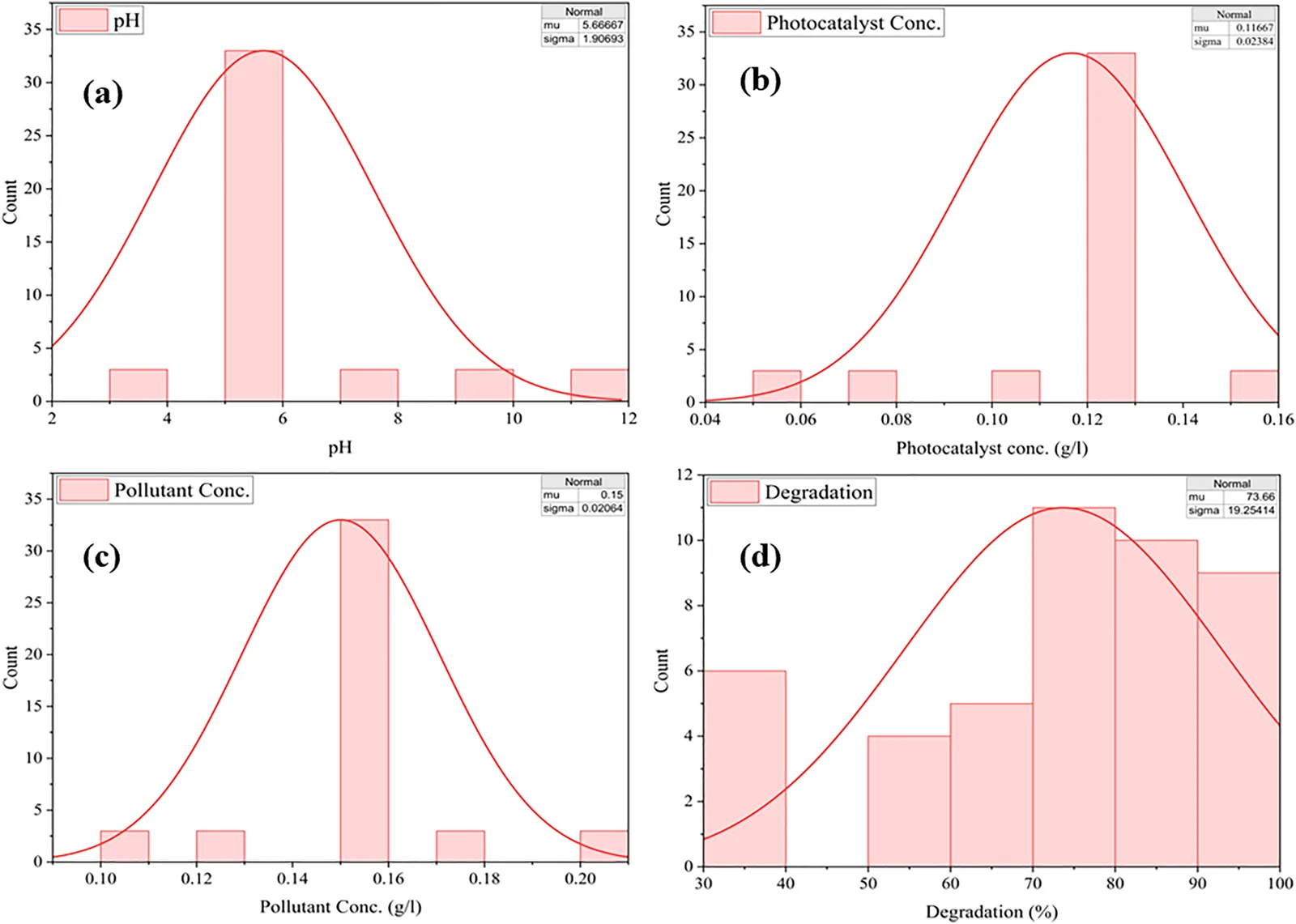
Distribution histograms of input variables; (a) pH, (b) photocatalyst conc. (c) pollutant conc. and output variable (d) pollutant degradation used in this study.


In machine learning, a high-quality dataset is crucial to ensuring the accuracy and flexibility of the generated model. A total of 45 chosen experimental outcomes were carried out. To improve the dependability and authenticity of the input dataset, original records with the same input parameters but different output values were averaged before being employed in the ML model. To establish the dataset, 16 unique data points were derived from the 45 experimental results. This study selected three (3) attributes related to photocatalyst activity, i.e., reactor solution pH value, photocatalyst concentration, and pollutant concentration, as an input parameters. The histograms of the variables used in this study are presented in Fig 2, and the descriptive statistics are stated in Table 1. Fig 2 illustrates the distributions of all parameters, with the curved lines denoting normal distribution curves derived from each parameter’s mean (μ) and standard deviation (σ). The parameters of these normal distributions are clearly labeled in the figure with the standard notation N(μ, σ^2^). All input parameters were normalized employing Eq. (1); X represent the original data for each parameter, while μ and σ correspond to the mean and standard deviation of that parameter, respectively. This procedure eliminates the impact of each parameter’s scale and dimensionality on model training by transforming the original data into new data that has a mean of zero and a unit standard deviation. It is important to point out that, in order to avoid premature information leakage from the testing set to the XGBoost model, the Standard Scaler was trained exclusively on the training set and applied to standardize both the training and testing sets.

**Table 1:** Descriptive statistics of variables used in model development.

Descriptive statistics	Photocatalyst Conc.	Pollutant Conc.	pH	Degradation
Mean	0.117	0.150	5.667	73.660
Standard Error	0.004	0.003	0.284	2.870
Median	0.125	0.15	5	75.9
Mode	0.125	0.15	5	74.2
Standard Deviation	0.0238	0.0206	1.9069	19.2541
Sample Variance	0.0006	0.0004	3.6364	370.7220
Kurtosis	2.6257	2.4997	2.6257	−0.4905
Skewness	−1.738	0.000	1.738	−0.630
Range	0.1	0.1	8	64.8
Minimum	0.05	0.1	3	34.2
Maximum	0.15	0.2	11	99
Sum	5.25	6.75	255	3314.7
Count	45	45	45	45
Confidence Level (95.0%)	0.007	0.006	0.573	5.785

$$\begin{array}{c} X_{stand}=\frac{X-\mu }{\sqrt{\sigma^{\text{2}}}} \end{array}$$ (1)

**Fig 3:**
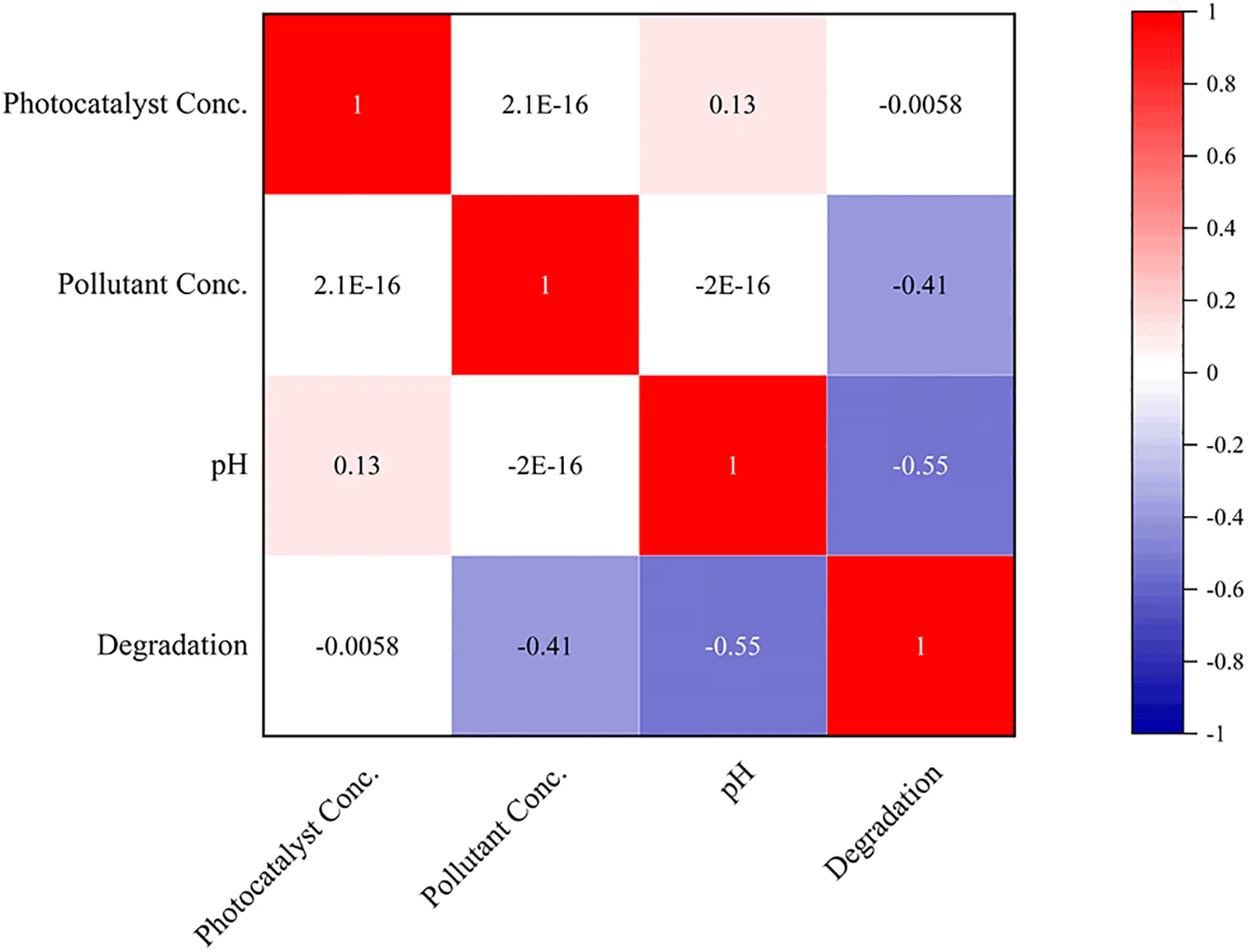
Pearson’s analysis of the variables involved in this study.

In order to assess the importance of input variables, Pearson’s correlation analysis was conducted, and the results are shown in Fig 3. It can be observed that pH and WO_3_ concentration depict a negative correlation towards the degradation rate. The parameters examined in this research show weak linear correlations. This indicates complicated internal nonlinearity and highlights the necessity of developing an advanced machine learning model to grasp complex relationships.

### 3.2. eXtreme Gradient Boosting (XGBoost) model overview

**Fig 4:**
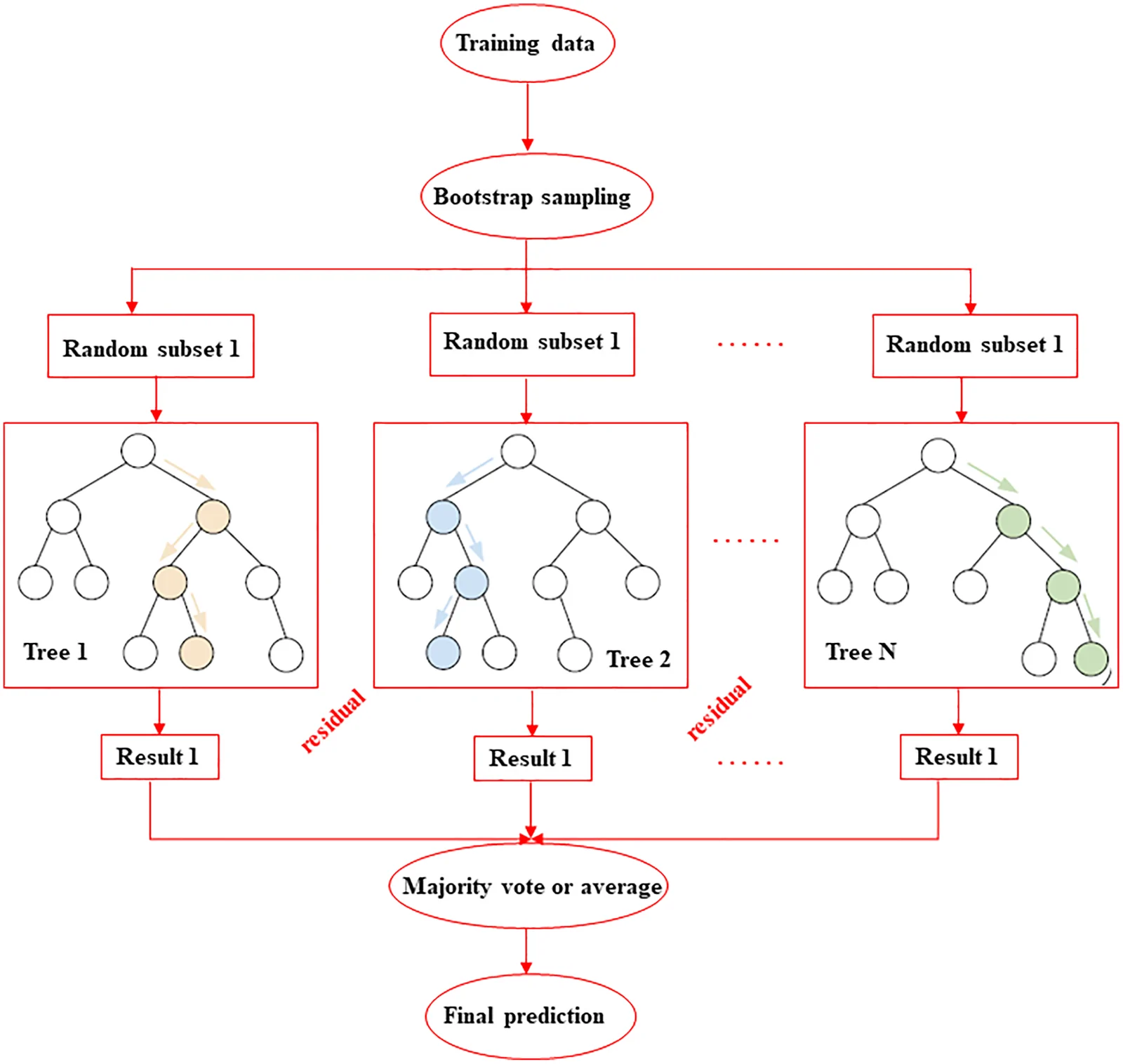
Structure of XGBoost model.

Chen and Guestrin introduced XGBoost, an open-source machine-learning library, in 2016 [27]. It stands out as a robust, adaptable, and easily transportable tool. In the context of supervised learning, it outperforms in dealing with regression, classification, and ranking issues. XGBoost is widely regarded by specialists in machine learning and data science as a reliable and effective algorithm, especially for handling structured/tabular data, particularly in cases with limited sample sizes. In addition, robustness to multicollinearity, and ability to capture nonlinear relationships. XGBoost ML model utilizes the bagging strategy, as shown in Fig 4, that is usually made up of numerous decision trees [28]. These trees are built by randomly choosing subsets of both the training data and features throughout the training phase. This method boosts the model’s diversity. The final prediction is derived through a majority vote for classification tasks or averaging for regression tasks, thereby improving the model’s accuracy and steadiness. It has been revealed that XGBoost can attribute higher weight to factors that earlier decision trees might have misestimated. XGBoost builds strong, reliable models by combining these various classifiers and predictors. Moreover, it also offers an intrinsic assessment of feature significance and can be analyzed using SHAP analysis [29]. This makes it an understandable model that sheds light on the explicit impact of different elements, a crucial feature for practical applications. However, the XGBoost model also has certain limitations. In particular, it involves numerous hyperparameters (e.g., maximum depth, subsampling), and identifying the optimal combination can be time-consuming and computationally expensive. Additionally, because it builds trees sequentially, it may overfit the data especially in the presence of noise, although regularization can mitigate this at the cost of careful tuning. Furthermore, despite its optimizations, training can still be slow and resource-intensive, particularly for large datasets or when extensive hyperparameter tuning is required.

### 3.3. Model building

**Fig 5:**
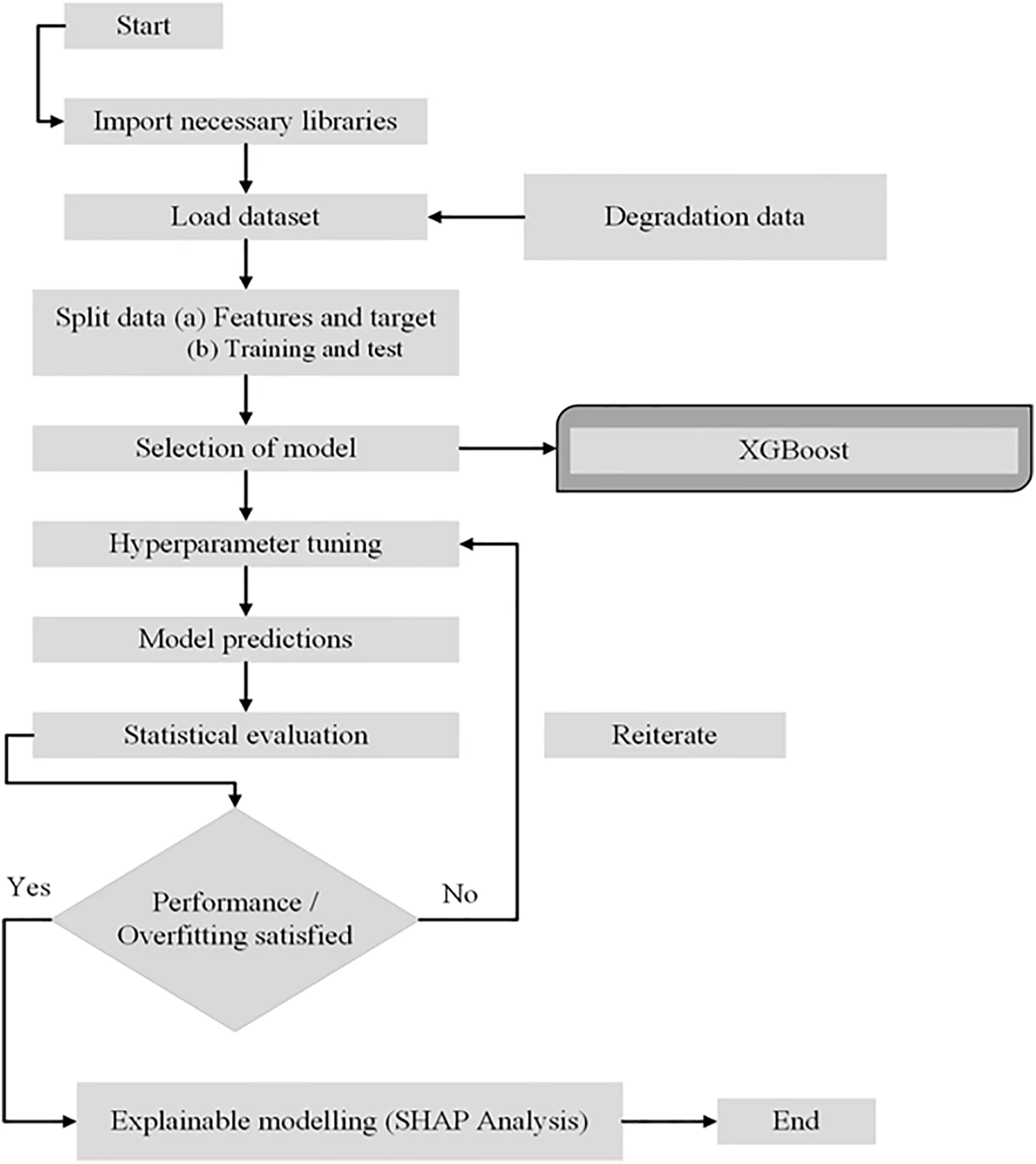
Flowchart of XGBoost model development (adopted from [30]).

This section describes the development of an XGBoost model to predict photocatalyst performance based on the parameters (pH, photocatalyst concentration, and pollutant concentration) studied. It is worth noting that the XGBoost model was chosen for its demonstrated predictive performance and interpretability. Fig 5 outlines a machine learning workflow that begins by preparing the data and breaking it into training and testing sets. The core process involves selecting the XGBoost model and iteratively tuning its hyperparameters, making predictions, and statistically evaluating the results. This loop continues until performance is satisfactory and overfitting is minimized. Finally, to ensure transparency and trust, the model’s decisions are explained using SHAP analysis.

To thoroughly assess the ML model, three assessment metrics were employed; Mean Absolute Error (MAE), Root Mean Squared Error (RMSE), and the coefficient of determination (R²). Eq.’s (2–4) describe the calculation techniques for these criteria, with ỳ_a_ indicating the projected value, y_a_ representing the experimental value, and n denoting the samples total number. In the context of model assessment, smaller MAE and RMSE values reflect superior predictive performance [17], whereas an R^2^ value nearer to 1 indicates greater prediction accuracy. Furthermore, it is essential to judge the difference in assessment criteria used for the training and testing sets. Considerable differences usually indicate overfitting, which must be tackled via hyperparameter optimization to improve generalizability.


$$\begin{array}{c} MAE=\;\frac{\text{1}}{n}\sum_{a=\text{1}}^{n}\left|{\overset{`}{y}}_{a}-\;y_{a}\right| \end{array}$$ (2)

$$\begin{array}{c} RSME=\;\sqrt{\frac{\text{1}}{n}}\;\sum_{a}^{n}{\left({\overset{`}{y}}_{a}-\;y_{a}\right)}^{\text{2}} \end{array}$$ (3)

$$\begin{array}{c} R^{\text{2}}=\text{1}-\;\sum_{a}^{n}{\left(y_{a}-{\overset{`}{y}}_{a}\right)}^{\text{2}}/\sum_{a=\text{1}}^{n}{\left(y_{a}-\overset{―}{y}\right)}^{\text{2}} \end{array}$$ (4)

### 3.4. SHAP inspection

The SHAP analysis offers a reliable approach to explain machine learning models [31]. In SHAP analysis, the machine learning model produces a predicted value for every data point based on all input parameters [17], and SHAP values are determined using Eq. (5), where y_a_ indicates the predicted value for the a^th^ sample, y_base_ represents the base value (usually the mean of the predicted variable), and f(x_a,j_) is the SHAP value that quantifies the influence of the j^th^ parameter on the anticipated value for the a^th^ sample. If f(x_a,j_) is positive, this means that the j^th^ parameter enhances the predicted value of the a^th^ sample; if it is negative, this signals a decline. The absolute value of f(x_a,j_) indicates the degree of the impact.


$$\begin{array}{c} y_{a}=\;y_{base}+\;{f(x}_{a,\text{1}})+\;{f(x}_{a,\text{2}})+\ldots +\;{f(x}_{a,j})+\ldots +\;{f(x}_{a,k}) \end{array}$$ (5)

### 3.5. XGBoost model effectiveness

**Fig 6:**
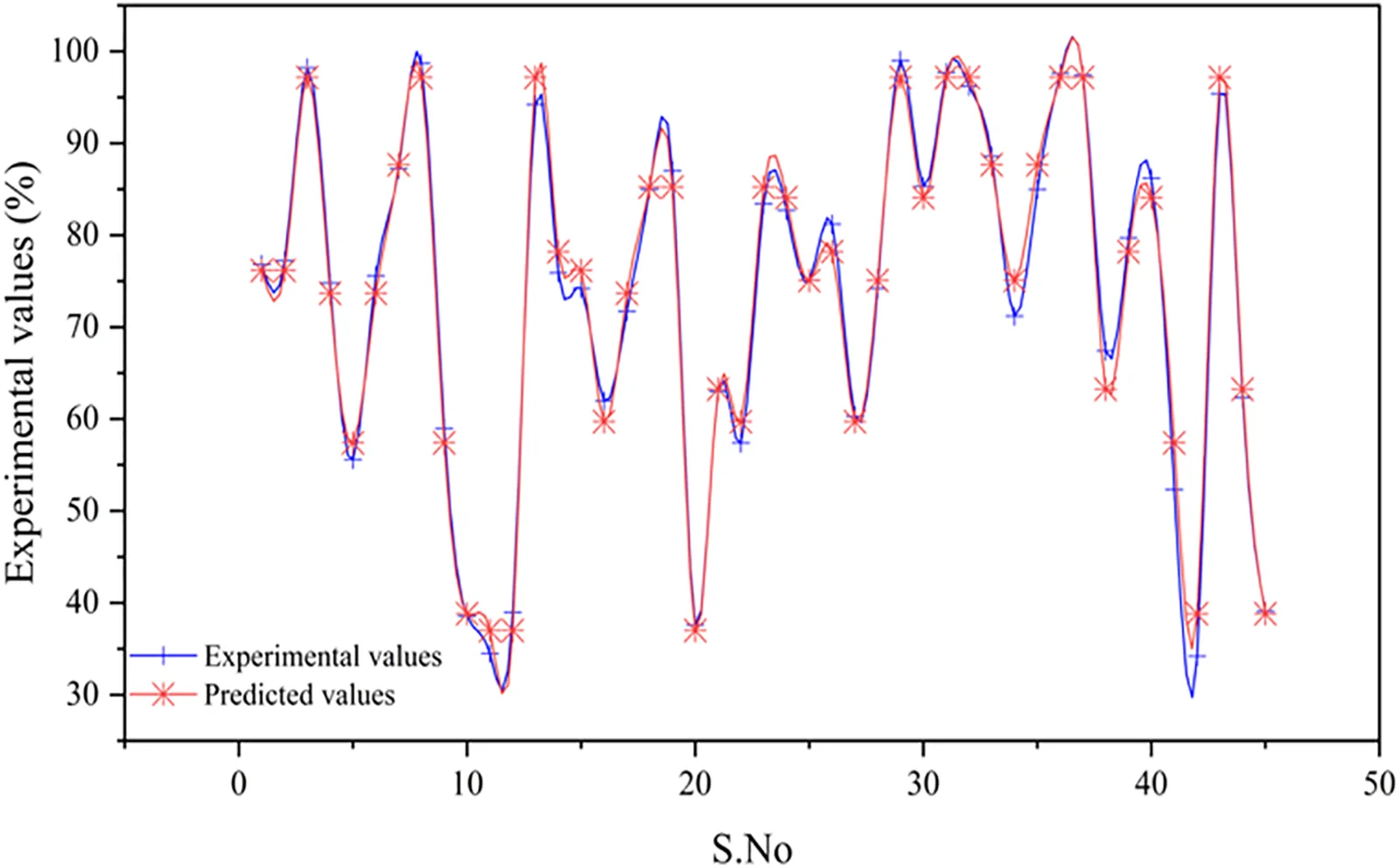
Tracing of experimental results by the predictions obtained from XGBoost model.

**Fig 7:**
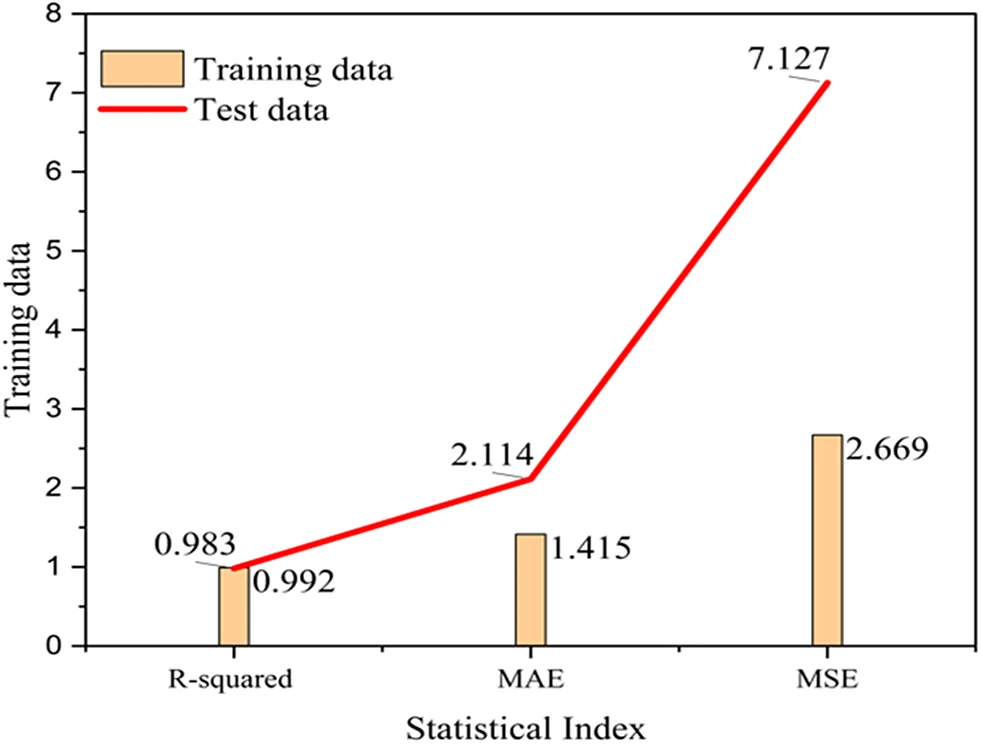
Statistical evaluation of XGBoost model.

The model was developed using 45 observations from experimental work by the author. According to Babanajad et. al., [32], a minimum object-to-parameter ratio of 5 is required to ensure the reliability of the final models. In the present study, this ratio is 45/3 = 15, which significantly exceeds the minimum requirement, as illustrated in the previous studies [33]. The performance of XGBoost model is illustrated in the form of tracing of experimental results by the prediction as presented in Fig 6. The data points are very tightly grouped to the experimental values. The performance on the unseen test data is also excellent. Fig 7 shows the performance of the XGBoost model utilizing the assessment metrics R^2^, MAE, and RMSE. The XGBoost model achieved great accuracy, with R^2^ values of 0.983 for the training set and 0.992 for the test set. The MAE values for the training and testing sets were 1.415 and 2.114, respectively, suggesting that the average forecasted errors were only 1.9% and 2.86%. The RMSE values for the training and testing sets were 2.6679 and 7.127, respectively. This further confirms the XGBoost model’s strong predictive performance. Moreover, differences in evaluation metrics between the training and test sets are essential for judging the model’s performance. The minor differences in the values of 0.009 for R^2^, 0.699 for MAE, and 4.458 for RMSE demonstrate the XGBoost model’s strong generalization capacity and minimal overfitting. The model good results in prediction and generalization highlight the dataset’s robustness and adequacy. The dataset’s strong predictive performance indicates that it presents a comprehensive depiction of the underlying patterns, making it well-suited to tackling the complexities of the problem.

To benchmark the performance of the proposed XGBoost model, Random Forest (RF) regressor was additionally implemented using the same dataset and train/test split (70%/30%, random state = 12). The RF model was optimized via grid search with 5-fold cross-validation, tuning key hyperparameters including the number of trees (n_estimators), maximum depth (max_depth), minimum samples per split and leaf, maximum features, and bootstrap option. The best configuration consisted of bootstrap = False, max_depth = 10, max_features = ’sqrt,’ min_samples_leaf = 1, min_samples_split = 2, and n_estimators = 50. Model performance was evaluated using the R^2^, MAE, and MSE on both training and testing subsets. As summarized in Table 2, the optimized RF model achieved excellent predictive accuracy, with a test R^2^ of 0.985, MAE of 2.023, and MSE of 6.437. On the training set, the RF model obtained an R^2^ of 0.992, MAE of 1.393, and MSE of 2.643, indicating minimal overfitting. Compared to XGBoost, Random Forest produced slightly lower test R^2^ (0.985 vs. 0.992) and higher test MAE (2.023 vs. 1.415) and MSE (6.437 vs. 2.669). While both models demonstrate strong generalization, XGBoost shows superior performance on the test data across all metrics, justifying its selection as the primary predictive model. Nevertheless, the RF results confirm that ensemble tree-based methods are highly suitable for this dataset, and the benchmarking addresses the need for comparison with alternative machine learning approaches.

**Table 2:** Performance comparison between XGBoost and Random Forest.

Model	Dataset	R^2^	MAE	MSE
XGBoost	Training	0.983	2.114	7.127
	Test	0.992	1.415	2.669
Random Forest (RF)	Training	0.992	1.393	2.643
	Test	0.985	2.023	6.436

### 3.6. SHAP analysis

**Fig 8:**
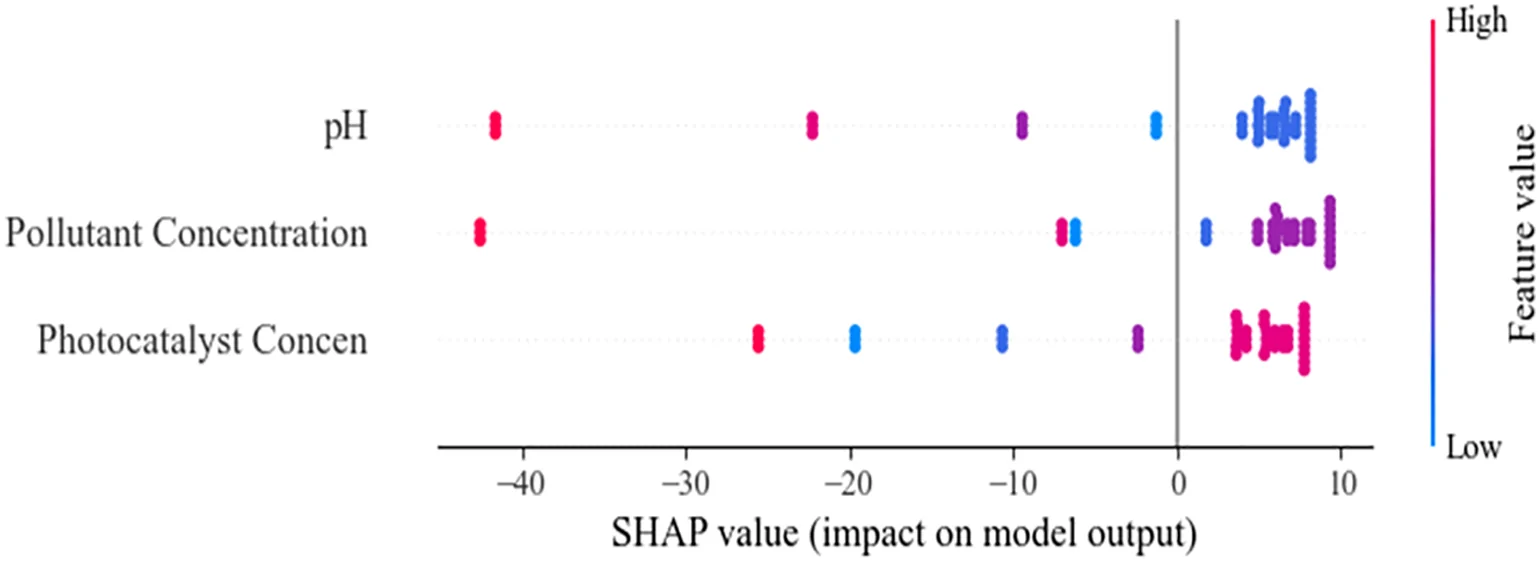
Summary plot of the SHAP analysis.

**Fig 9:**
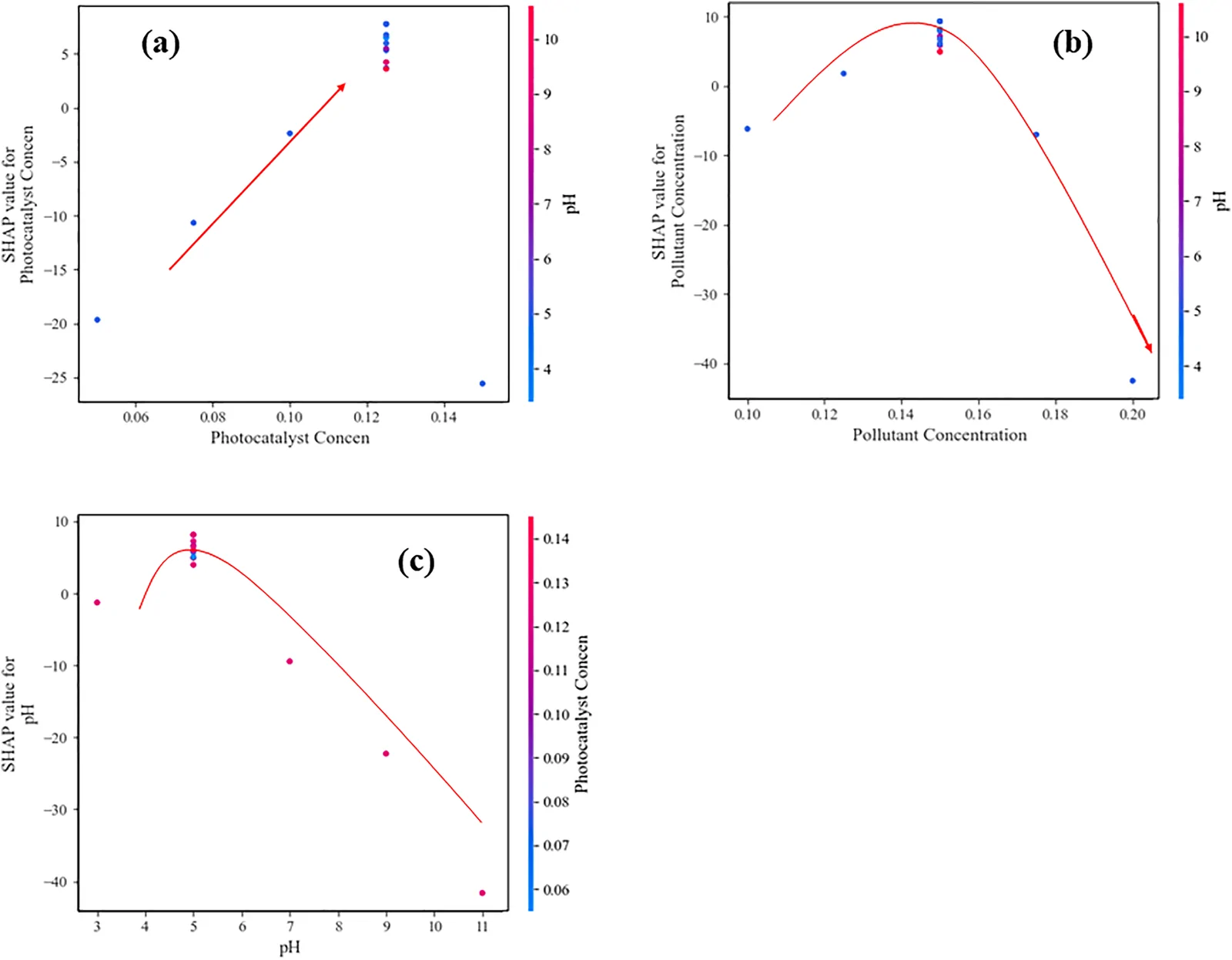
Dependence plot of variables.

This section illustrates the use of SHAP analysis to explain the XGBoost model’s predictions and assess the impact of all input variables on degradation. It is important to be illustrated that in SHAP analysis, the SHAP values measure each parameter’s contribution to the final prediction compared to the baseline (average value). In this work, the baseline is the average photocatalytic activity of 73.66% as shown in Table 1. Fig 8 presents the summary plot from the SHAP analysis, with the vertical axis denoting the SHAP value and the color scale on the right representing the magnitude of the parameter value. The parameters are ordered according to their mean absolute SHAP values, reflecting their relative importance in influencing photocatalytic activity. From top to bottom, significance of the parameters decreases, with pH value being identified as the most significant factor for forecasting photocatalytic activity. The primacy of pH in the SHAP ranking has a well-grounded physicochemical basis. The surface charge of the photocatalyst is strongly pH-dependent; at pH values near or below the point of zero charge, the catalyst surface becomes positively charged, which promotes electrostatic attraction of anionic pollutant molecules and enhances adsorption, a prerequisite step for effective photocatalytic degradation. Furthermore, pH governs the generation and speciation of reactive oxygen species (ROS): under acidic to mildly acidic conditions, hydroxyl radical (OH^•^) production via valence band holes is thermodynamically favored, whereas at strongly alkaline pH, competing reactions such as hole scavenging by excess OH**^−^** can paradoxically reduce net degradation efficiency despite an apparent abundance of hydroxyl donors. Pollutant concentration is the next most important parameter, while catalyst dosage has a comparatively smaller effect. The dependence plots shown in Fig 9 demonstrate a linear increase in decomposition with increasing photocatalyst concentration. The degradation plummets around 0.14–0.16 of pollutant concentration and a pH value of 5. The subsequent sections will provide a more detailed discussion of the effect of the parameters studied.

## 4. Experimental results and discussion

### 4.1. Influence of operational parameters

#### 4.1.1. Influence of pH.

**Fig 10:**
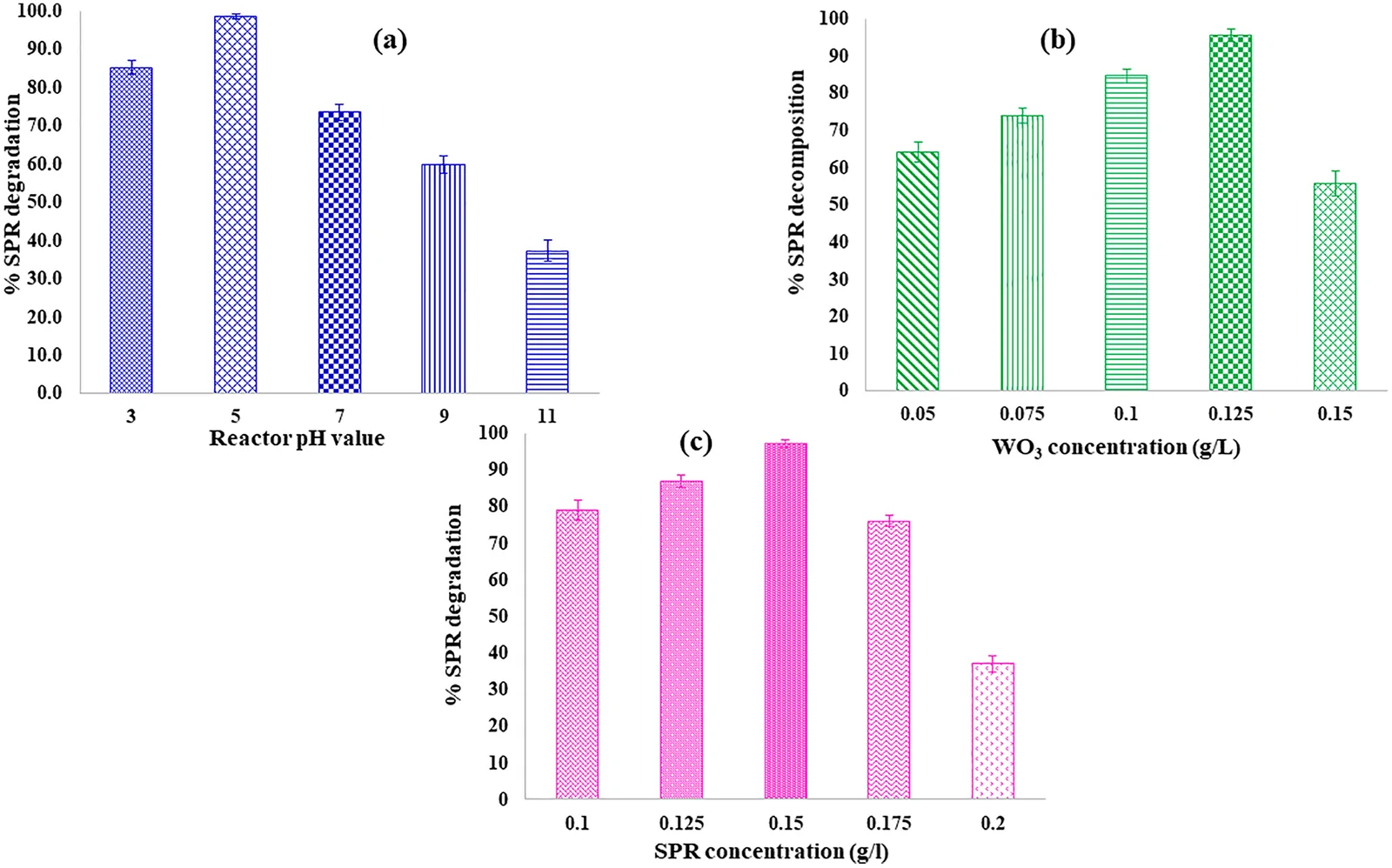
Influence of (a) Reactor solution pH value, (b) WO_3_ concentration and (c) SPR concentration on photocatalytic pollutant degradation.

The influence of initial pH values between 3.0 and 11.0 on SPR decomposition WO_3_/UV light system was examined under optimal content of WO_3_ (0.125 g/l), and the results presented in Fig 10(a). It is important to note that strongly acidic conditions (pH 3–7) greatly enhanced the elimination of SPR on the photocatalyst compared to alkaline conditions (pH > 7–11). The pH level of the SPR solution (0.15 g/l) was found to be 5.8 and the measured pollutant decomposition was 80.2%. The observed activity trend was 85%, 98.7%, 74.2%, 60.3%, 38.6% at pH value of 3, 5, 7, 9 and 11, respectively. It is reported elsewhere [34] that the pH of the reactor suspension has a major impact on the photocatalytic decomposition of contaminant because it alters the chemical structure of the organic molecules in the solution as well as the surface charge of the nanocomposite. SPR has a pKa value of 7.9 [35] and the solution pH value determines whether the amino and OH^-^ group are protonated or deprotonated. SPR will be positively charged if the solution pH value is less than its pKa value and vice-versa. The PZC (point zero charge) value for WO_3_ powder was determined to be 3.0 “S1 Fig.”, implies that the photocatalyst surface carries a negative charge at solution pH levels above 3.0 and a positive charge at pH levels below 3.0. Hence, the optimal pH value for photocatalysis lies within ${pH}_{PZC}^{{WO}_{\text{3}}}>pH<{pKa}^{SPR}$ where SPR molecules are positively charged due to protonated amino groups and should easily attract the negatively charged WO_3_ particles. In acidic conditions, the electrostatic attractions between SPR molecules and the nano WO_3_ catalyst surface intensify, resulting in an increased rate of pollutant adsorption on the catalyst surface and an enhanced decomposition rate of SPR. Another factor contributing to improved SPR decomposition efficiency at lower initial pH levels is the high concentration of H^+^ ions in acidic environments. This concentration leads to the production of hydroxyl (OH^-^) ions through reactions with dissolved oxygen in the solution, forming superoxide radicals that eventually convert into OH^•^ radicals, which results in the decompaction of pollutant molecules [36]. Moreover, in alkaline environment the decreased decomposition efficiency may be due to the electrostatic repulsive interaction between WO_3_ nano powder negatively charged surface and the negatively charged, deprotonated SPR molecules. This results in the decreased adsorption of contaminant molecules on WO_3_ photocatalyst surface. In addition, the hydroxyl radicals may be scavenged and prevented from reacting with contaminating molecules at high solution pH values. The observed results are supported by findings reported in the literature [37,38]. To sum up, the photocatalytic degradation of SPR is highly influenced by the pH level of the solution; acidic conditions accelerate the breakdown rate by increasing the electrostatic attraction between the pollutant molecules and nano-material surface and promotes the production of OH^•^ radicals. Developing more efficient photocatalytic procedures for the removal of different environmental contaminants can be aided by an understanding of these principles.

#### 4.1.2. Influence of photocatalyst dosage.

The process of photocatalytic degradation involves a semiconductor oxide absorbing light with appropriate energy to break down complex and stable molecular structures into non-poisonous and lighter molecules having lower molecular weight. The process is efficient and cost-effective and can be used to convert light energy into chemical energy in the form of exciton pairs (e^-^/h^+^), which are then employed directly or to produce redox anions and radicals for the degradation of organic compounds such as antibiotics. To determine the optimum photocatalyst dosage the model antibiotic degradation tests with an initial concentration of 0.15 g/l were performed. The degradation process duration was set as 60 min, while the photocatalyst content in successive experiments was varied from 0.05 to 1.5 g/l. As the amount of WO_3_ increases, the number of active sites on its surface rises, leading to a corresponding increase in the photocatalytic reaction rate. The observed percentage degradation rate as illustrated in Fig 10(b) was 63.2, 75.6, 85.3, 95.4 and 59.0 for 0.05, 0.075, 0.1, 0.125 and 0.15 g/l WO_3_ dosage, respectively. This implies that the decomposition rate of SPR increases when the WO_3_ content increases from 0.50 to 1.25 g/l. Afterwords further increase in WO_3_ concentration leads to a decrease in the decomposition rate of SPR. This behavior may be ascribed to limitations imposed by the reactor geometry and operating conditions, as well as a reduction in the effective exposed surface area of WO_3_. A certain increase in concentration can boost the production of OH^•^ radicals from illuminated WO_3_ to pollutant degradation or in other words as photocatalyst concentration increases as a consequence of many active sites for the photocatalytic reaction are available. But it is important to remember that this relationship is unlikely to be linear, as an optimal dosing range appears to exist. These findings imply that excessively high dosages (>1.25 g/l) do not guarantee better results, and the optimum catalyst dosage lies within the range of 0.5–1.25 g/l. The possible explanation lies in the equilibrium between a greater number of catalytic active sites and the possibility of catalyst particle aggregation that comes with higher dosages [39], which might reduce overall efficiency on decreasing the number of surface active sites, moreover, high catalyst loading causes increase in opacity and light scattering thereby limiting light penetration through the sample and ultimately decreasing overall efficiency [40]. In any given application, this optimum WO_3_ concentration is needed in order to prevent unnecessary and guarantee complete absorption of radiation photons for effective degradation.

#### 4.1.3. Influence of SPR concentration.

Experiments were conducted to study the variation in the degradation percentage at different SPR concentration ranging from 0.1 to 0.2 g/l at the optimum reactor pH of 5.0 and WO_3_ content of 0.125 g/l. It could be observed from Fig 10(c) that decomposition rate appeared to be improved on increasing SPR concentration up to 0.15 g/l, further increase in model pollutant concentration causes a decrease in degradation rate. This implies that a greater density of pollutant molecules increases the chances for interaction with WO_3_ photocatalyst particles. There is an optimal concentration level of 0.15 g/l, beyond this point, further increases in concentration lead to a decrease in efficiency. This may be attributed to the saturation of active sites on the surface of the catalyst [41], which retards photocatalytic activity. In addition, an increase in pollutant molecules hinders light transmittance and the photon transmission path, thereby obstructing the migration of photons to the active site of the photocatalytic process. Furthermore, as the SPR concentration increases, the number of adsorbed SPR molecules on the WO_3_ surface increases. The formation of h^+^, OH^•^ radicals, and other oxidants on the photocatalyst surface remains constant, leading to a decrease in the relative number of reactive species available to attack SPR molecules. Additionally, the formation of more intermediates creates competition between reactive radicals and intermediates for active reaction sites. As a result, the overall decomposition efficiency decreases. For environmental remediation processes to achieve optimal photocatalytic degradation effectiveness, the initial pollutant concentration must be optimized.

### 4.2. Effect of scavengers

**Fig 11:**
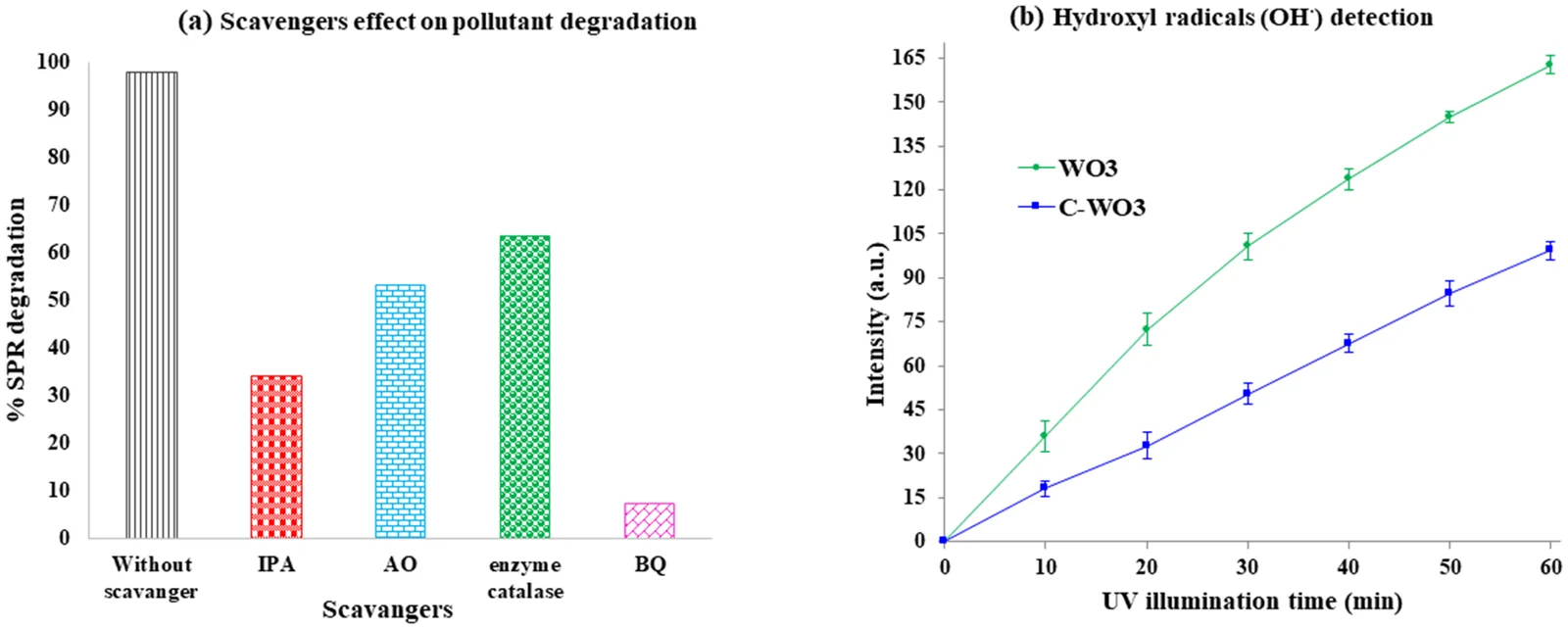
(a) Role of scavengers on model pollutant degradation, (b) variation in PL intensity of the dispersed TA solution under UV light with as-prepared and commercial WO_3_ photocatalysts.

In order to gain a better understanding of the mechanisms involved in SPR degradation over the WO_3_ photocatalyst, experiments were conducted to identify the reactive species that contribute to the photocatalytic process. Isopropyl alcohol, enzyme catalase, benzoquinone, and ammonium oxalate were employed as scavengers for hydroxyl radicals (OH^•^), hydrogen peroxide (H_2_O_2_), superoxide anions (O_2_^•−^) and hole (h^+^), respectively. Fig 11(a) illustrates the effect of these scavengers on the SPR decomposition rate. Without adding scavengers, the WO_3_ photocatalyst reached a SPR decomposition efficiency of 97.8%. However, with the addition of isopropyl alcohol, a substantial decrease in SPR decomposition was noted (34.1%), indicating that OH^•^ radicals were the main reactive species involved in the decomposition process. Moreover, a 53.3% and 63.5% SPR removal rate were attained by adding ammonium oxalate and enzyme catalase, respectively, this shows that h^+^ and H_2_O_2_ also played a role in degrading pollutant species. In contrast, the presence of benzoquinone led to only a minor reduction in the SPR removal rate (7.4%). This finding portrays that O_2_^•−^ species were not formed efficiently during the decomposition process. These findings show that OH^•^ radicals, which are produced by excited holes (h^+^s), are essential for the photocatalytic breakdown of SPR. This implies that OH^•^ radicals are extremely reactive and efficient at breaking down organic contaminants, it is crucial to optimize photocatalytic systems to maximize their generation. Furthermore, the contribution of holes and hydrogen peroxide should not be overlooked, as they also contribute significantly to the overall efficiency of the photocatalytic process. To further demonstrate the enhanced production of OH^•^ radicals by the prepared photocatalyst, a PL technique [42] was employed using terephthalic acid (TA) as the fluorescent probe to track the production of hydroxyl radicals. When TA reacts with OH^•^ radicals (${OH}^{\cdot }+TA\to TAOH)$ a highly fluorescent 2-hydroxy terephathalic acid is produced. The TAOH fluorescence intensity as a function of UV light in the suspension solution with the as-synthesized catalyst and for comparison with commercial WO_3_ (SkySpring nano materials, Inc., USA, purity > 99.5% light yellow nano powders, monoclinic crystal structure, average particle size < 100nm, specific surface area 5–8 m^2^/g, pore diameter 1.8 nm, anisotropic morphology and band gap 2.62 eV [43]) at pH 5.0 is displayed in Fig 11(b). It can be observed that the as-synthesized WO_3_ formed more hydroxyl radicals compared to the commercial WO_3_ as consequence these reactive species (OH^•^) will actively contribute to its higher activity against the SPR molecules.

### 4.3. Photocatalytic activity tests under optimum conditions

**Fig 12:**
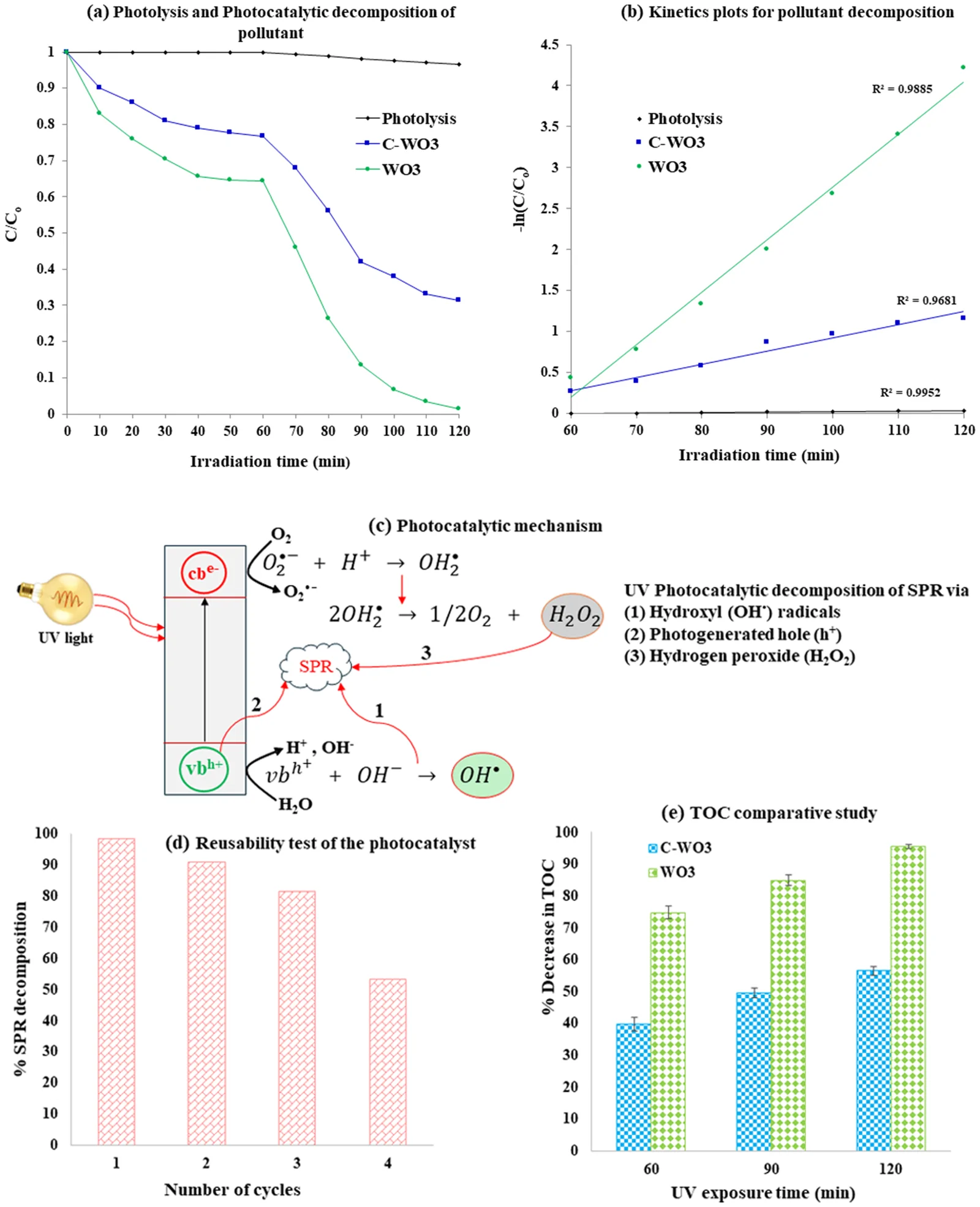
(a) Model SPR pollutant adsorption and degradation in the presence and absence of UV light, (b) plot of −ln(C/C_o_) versus UV irradiation time, (c) schematic illustrating the UV photocatalytic mechanism, (d) recyclability of WO_3_ photocatalyst under optimum condition against SPR model pollutant and (e) total organic carbon analysis of the annealed as-prepared WO_3_ and C-WO_3_ materials.

Fig 12(a) depicts the photocatalytic decomposition of spiramycin (SPR) under optimum conditions of reactor solution pH value of 5.0, photocatalyst dosage of 0.125 g/l, pollutant concentration of 0.15 g/l after 1 hr of exposure to UV light. A degradation efficiency of 98.6% was achieved with a significant contribution from adsorption in the absence of light irradiation. Before the light irradiation, the catalytic solution was stirred magnetically for one hour in the dark to achieve adsorption equilibrium. Moreover, we confirm that without a WO_3_ photocatalyst, i.e., photolysis, the SPR concentration was almost invariable, which proved that the pollutant decomposition is caused entirely by the photocatalytic reaction. In addition, for comparative analysis, commercial WO_3_ was also evaluated in the activity tests, it was evidently observed that the as-prepared WO_3_ exhibited higher activity than the commercial WO_3_. This result further portrays that both photocatalyst and light are needed for the decomposition process. The enhancement in catalytic activity by the as-prepared material can be attributed to the reduced crystallite size via greater surface area compared to commercial WO_3_. Photocatalysis is a surface phenomenon, larger surface area with porous structure results in large active sites for the adsorption of the contaminant species which are then decomposed by the redox species (OH^•^, H_2_O_2_) generated by the photo produced charge carriers (e^-^/h^+^). The good crystallinity of as-prepared WO_3_ powder (evident from the high intense and sharpe XRD peaks [24]) may also increase its activity, as higher crystallinity reduces structural defects. In contrast, poorly crystalline powders (less intense XRD (002) and (004) reflections at 23.1° and 47.2°, respectively [43]) contain more defects, which act as recombination centers for photogenerated charge carriers, thereby decreasing photocatalytic performance. Additionally, materials with a high degree of crystallinity exhibit better charge carriers production, mobility and lifetime of separated charges, charge migration, and trapping processes at the solution–particle interface [44]. Moreover, the as-synthesized WO_3_ material has a uniform needle like morphology in comparison to the polydisperse nature of commercial WO_3_. This further portrays that the sharp corners of the as-synthesized WO_3_ may be beneficial towards higher photocatalytic activity, atoms located at edges and corners possess lower coordination numbers, which generally makes them more catalytically active [45]. In conclusion, when exposed to UV light, the synthesized nanocomposite demonstrated better photocatalytic performance for SPR degradation than commercial WO_3_ nanoparticles. This finding demonstrates the potential of prepared WO_3_ nanoparticles for environmental cleanup and the significance of maximizing efficiency through photocatalyst composition optimization.

The Langmuir Hinshelwood (L-H) kinetic model is frequently used for the photocatalytic decomposition of organic pollutants (in our case spiramycine, SPR) in solutions. The L-H model formulation for an ideal constant volume batch reactor that contains the model pollutant is


$$\begin{array}{c} -\frac{dC}{dt}=\frac{kK^{\prime }C^{SPR}}{\text{1}+K^{\prime }C^{SPR}} \end{array}$$ (6)

where, k is the reaction rate constant (mg/l min), K′ is the equilibrium constant (L/mg) of the adsorbed molecules (SPR) on the surface of the photocatalyst at the reaction temperature and C^SPR^ is the concentration (mg/l) of the decomposed SPR molecules in solution. Eq. (6) becomes


$$\begin{array}{c} -\frac{dC}{dt}=\;\frac{k^{app}C^{SPR}}{\text{1}+K^{\prime }C^{SPR}} \end{array}$$ (7)

k^app^ is the apparent reaction rate constant (min^−1^) equal to kK′. At low concentration, K′C^SPR^ <<1, then Eq. (7) simplifies into the following first order equation:


$$\begin{array}{c} -\frac{{dC}^{SPR}}{dt}=\;k^{app}*\;C^{SPR} \end{array}$$ (8)

The kinetic plots of SPR degradation are shown in Fig 12(b), which fits the given experimental data using pseudo first-order kinetics. Assuming C^SPR^ = 0, when t = 0, integrating Eq. (8) gives;


$$\begin{array}{c} n\frac{C}{C_{O}}=\;k^{app}*t \end{array}$$ (9)

where C_o_ is the SPR initial concentration at time, t = 0, C is SPR concentration at any time (t) during the photocatalytic reaction. The k^app^ values were calculated from the slopes of the linear graphs plotted for −ln(C_o_/C) against t, and the observed trend was WO_3_ > C-WO_3_. The high correlation coefficient (R^2^) value depicted in Fig 11(b) portrays a good match to a first order kinetic reaction. The corresponding k^app^ value for WO_3_ (0.064 min^-1^) is significantly greater than commercial WO_3_ (0.016 min^-1^), indicating high photodecomposition performance of prepared and optimized material.

The probable photocatalytic reaction mechanism for the decomposition of SPR assisted with WO_3_ under UV irradiation is presented in Fig 12(c). Upon UV light e^-^s and h^+^s are produced in WO_3_ conduction band (cb) and valance band (vb), respectively ($h\vartheta +\;{WO}_{\text{3}}\;\to \;{vb}^{h^{+}}+\;{cb}^{e^{-}}).$ The WO_3_ vb h^+^s has high oxidation power (E_vb_ = + 3.1–3.2 V_NHE_) which facilitate the water oxidation ($E_{H_{\text{2}}O/O_{\text{2}}}$ = + 1.23 $V_{NHE})$ and results in the formation of OH^•^ and H^+^ entities (${vb}^{h^{+}}+\;H_{\text{2}}O\;\to \;{OH}^{\cdot }+\;{OH}^{-}\;;\;{h^{+}}_{vb}+\;{OH}^{-}\;\to \;{OH}^{\cdot })$. The cb e^-^s react with dissolved or adsorbed oxygen to generate superoxide anions, ${(cb}^{e^{-}}+\;O_{\text{2}}\;\to O_{\text{2}}^{-\cdot })$. The superoxide anions (O_2_^-•^) via disproportionation with protons are converted into hydrogen peroxide $\left({cb}^{e^{-}}+H^{+}{+\;O}_{\text{2}}^{-\cdot }\to H_{\text{2}}O_{\text{2}}\right)$ or it take the form of HO_2_^•^ via protonation ${(H}^{+}+\;O_{\text{2}}^{-\cdot }\;\to \;{HO}_{\text{2}}^{\cdot }\;;\;{HO}_{\text{2}}^{\cdot }+\;{HO}_{\text{2}}^{\cdot }\;\to \;{\text{1}/\text{2}O}_{\text{2}}+\;H_{\text{2}}O_{\text{2}})$, whose lifespan is short due to the quick reaction with O_2_^•−^ or HO_2_^•^ species combine to form stable hydrogen peroxide. Subsequently, one electron reduction of hydrogen peroxide produces hydroxyl radicals (${cb}^{e^{-}}+\;H^{+}+\;H_{\text{2}}O_{\text{2}}\;\to \;H_{\text{2}}O+\;{OH}^{\cdot }).$ This implies that the generation of OH^•^ radicals occurs via two ways, reduction of oxygen and oxidation of hydroxyl ions and water molecules. It is reported that conduction band edge of WO_3_ is low (+0.5 V vs NHE @ pH = 0), produced electrons in WO_3_ conduction band cannot be effectively consumed by oxygen molecules via single electron reduction to produced O_2_^•-^ species further, however, the production and presence of oxidizing species are influenced by several factors, including the potential of the conduction band edge (E^cb^), the pH at the photocatalyst’s zero-point charge (pH_ZPC_), and the pH of the surrounding medium. For the $O_{\text{2}}/O_{\text{2}}^{\cdot -}$couple, an appropriate E^cb^ (i.e., reduction potential) is typically E^cb^ = −0.28 V_NHE_. A neutral or basic pH (≥ 7.0) promotes the generation of O_2_^•−^, whereas a lower pH (≤ 7.0) makes it easier for OH^•^ and H_2_O_2_ to form. Following the radicals quenching experiments, we observed that OH^•^ radicals were the primary species generated via oxidative paths which is further enhanced by the optimum lower pH value of the reactor photocatalytic solution. The decomposition of the model pollutant (SPR) can be attributed primarily to hydroxyl radicals due to their strong oxidizing ability, in addition SPR molecules are also decomposed by holes and hydrogen peroxide, in nutshell results in larger decomposition of SPR. The WO_3_ photocatalyst displayed a slight decrease in the SPR decomposition efficiency to 81.6% over the first three consecutive cycles, followed by a major decrease in the fourth cycle (53.4%), as illustrated in Fig 12(d). The reduction in photocatalytic performance may be attributed to a decrease in surface area resulting from the coagulation of nanoparticles and/or the buildup of reaction byproducts on the surface towards saturating some of the active sites, in addition material loss due to successive washing followed by oven drying, surface fouling or structural changes may also be the reasons for the decrease in recycle photocatalyst activity [46]. Overall, these recyclability tests reveal the strong stability of the as-prepared WO_3_ material even after consecutive usage and emphasize photocatalyst suitability for practical and sustainable environmental applications.

Photocatalytic degradation of pharmaceutical antibiotics is often accompanied by the formation of intermediate species, some of which may be more toxic than the parent compound. Therefore, monitoring total organic carbon (TOC) during the degradation process is essential for assessing mineralization efficiency, treatment effectiveness, and potential toxicity reduction, ensuring safety for both human health and the environment. TOC analysis experiments were conducted to track the mineralization of SPR using commercially available WO_3_ and as prepared WO_3_ under optimum process conditions (reactor solution pH value of 5.0, WO_3_ dosage of 0.125 g/l, SPR concentration of 0.15 g/l). As shown in Fig 12 (e), the percentage decrease in TOC increased progressively with UV exposure time. After 60 minutes of UV irradiation, TOC removal reached 74.8% for the as-synthesize WO_3_ photocatalyst, compared to 39.7% for commercial WO_3_. This improvement continued with longer irradiation times, achieving 84.9% (WO_3_) and 49.6% (C-WO_3_) after 90 minutes, and 95.7% (WO_3_) versus 56.6% (C-WO_3_) after 180 minutes, respectively.

The TOC analysis reveals that the usage of as-prepared WO_3_ may promote the formation of simpler and lower molecular weight intermediate species during the SPR photocatalytic degradation process compared to commercial WO_3_, which is consistent with the observed decomposition results. Similar observations have been reported in literature, where the mother compound transforms into different intermediate organic species, while a substantial fraction undergoes complete mineralization to carbon dioxide and water [47]. The produced intermediates may exhibit resistance to further degradation but may generally be less harmful and are not expected to pose significant environmental risks.

### 4.4. WO_3_ photocatalyst comparison for spiramycin degradation

The WO_3_ photocatalytic efficiency compared to other photocatalyst reported in the literature for the degradation of spiramycin is portrayed in Table 3. This is obvious that photocatalytic decomposition is a complex process in which the general efficacy of the organic contaminant removal is determined by the interaction of photocatalyst physiochemical properties (type, size, surface area, band gap, defect structures etc.), contaminant properties (type, dosage etc.) and process operating circumstances (reactor type and dimensions, reactor solution pH, light source, intensity and duration, reaction temperature of the system etc.). Therefore, a fair comparison is not possible, however, the photocatalytic activity of our prepared WO_3_ powder is very promising compared to the commercially available TiO_2_ and ZnO respectively.

**Table 3:** As-prepared WO_3_ photocatalytic performance for spiramycin (SPR) compared to published works.

Photocatalyst	BET surface area (m^2^/g)	Illumination time (min)	Spiramycin dosage (mg/l)	Photocatalyst content (mg/l)	% decomposition	Ref.
TiO_2_	4.61	360-UV^α^	100	250	90	[48]
ZnO	25.0	80-UV^β^	10	1000	97	[49]
P25	65	80-UV^γ^	0.03	100	91	[50]
WO_3_	99.0	60-UV	150	125	98.6	This work

αTwo UV (λ_max_ @ 365 nm) lamps and commercial TiO_2_ were supplied by BIOCHEM ChemoPharma (Quebec, QC, Canada), ^β^ 450 W Xenon arc lamp (LotOriel Group, Italy) and commercial ZnO were purchased from Sigma-Aldrich (Saint Louis, MO, USA). Photoreactor pH was 5.5 and ^γ^ Degussa P25 TiO_2_ (80% anatase and 20% rutile) was purchased from Evonik, Essen, Germany and 450 W Xenon arc lamp (λ < 320 nm, LotOriel Group, Italy) was used during photocatalysis.

## 5. Conclusion

Currently, research on the photocatalytic decomposition of antibiotics using WO_3_ nano-materials has made considerable progress; however, numerous shortcomings still need to be focused. For example, the present study focuses on the degradation of spiramycin (SPR) using as-synthesized WO_3_, along with the effects of key operational parameters (pH value, photocatalysts dosage and pollutant concentration) and machine learning modeling for parameters screening and process optimization. Under the parametric effects studied, experiments have shown that highest pollutant degrading was achieved (a) under a pH value of 5.0 (98.7%), (b) under a photocatalyst dosage of 0.125 g/l (94.2%) and under pollutant concentration 0.15 g/l (97.6%), respectively. For optimization purposes the studied data was modeled in a selected ML technique of XGBoost. The developed XGBoost model showed remarkable predictive performance SPR degradation, achieving R^2^ values of 0.983 and 0.992 on the training and testing sets, respectively. The two sets minimal R^2^ difference of 0.009 suggests a robust generalization capacity and minimal overfitting. The model’s robustness is further validated by additional metrics like MAE and RMSE, establishing it as a dependable tool for evaluating spiramycin (SPR) degradation. SHAP analysis revealed the primary influence of solution pH value on pollutant degradation, followed by pollutant concentration, while the effect of photocatalyst dosage was recorded the least. In addition, under optimum condition (pH (5.0), WO_3_ concentration (0.125 g/l) and SPR concentration (0.15 g/l), a maximum 98.6% pollutant degradation was achieved. Moreover, we also carried out scavenging experiments which give insight into the proposed mechanism for pollutant degradation under UV irradiation of WO_3_ material. It was observed that mainly OH^•^ radicals followed by photogenerated holes (h^+^s) and H_2_O_2_ were the key species that help in the decomposition process, also the photocatalyst recyclability was tested for four (4) cycles. In summary, this study successfully demonstrated the application of WO_3_ material for antibiotic degradation. It also proposed XGBoost machine learning model to predict pollutant degradation, which showed excellent predictive performance. The obtained results can serve as a reference and provide useful insights into further research and practical applications in this domain.

## Supporting information

S1 Fig Surface charge properties of as-synthesized WO_3_. Zeta potential measurement analysis was conducted by using a Malvern, Nano-ZS Zetasizer to determine the pH value at which the surface of the oxide (WO_3_) is uncharged [1]. In the studied pH range 2.0–5.0 the measured pH_ZPC_ of WO_3_ was 3.0. [1] H. Khan, M. Habibb, A. Khan, D.C. Boffito, A modified sol-gel synthesis to yield a stable Fe^3+^/ZnO photocatalyst: Degradation of water pollutants and mechanistic insights under UV and visible light, Journal of Environmental Chemical Engineering, 8 (2020) 1–12.
(TIF)

## References

[1] Santos LHMLM, Gros M, Rodriguez-Mozaz S, Delerue-Matos C, Pena A, Barceló D, et al. Contribution of hospital effluents to the load of pharmaceuticals in urban wastewaters: identification of ecologically relevant pharmaceuticals. Sci Total Environ. 2013;461–462:302–16. doi: 10.1016/j.scitotenv.2013.04.077 23732224

[2] Williams MR, Stedtfeld RD, Guo X, Hashsham SA. Antimicrobial Resistance in the Environment. Water Environ Res. 2016;88(10):1951–67. doi: 10.2175/106143016X14696400495974 27620115

[3] Klein EY, Impalli I, Poleon S, Denoel P, Cipriano M, Van Boeckel TP, et al. Global trends in antibiotic consumption during 2016-2023 and future projections through 2030. Proc Natl Acad Sci U S A. 2024;121(49):e2411919121. doi: 10.1073/pnas.2411919121 39556760 PMC11626136

[4] Oturan MA, Aaron J-J. Advanced Oxidation Processes in Water/Wastewater Treatment: Principles and Applications. A Review. Critical Reviews in Environmental Science and Technology. 2014;44(23):2577–641. doi: 10.1080/10643389.2013.829765

[5] Feng L, van Hullebusch ED, Rodrigo MA, Esposito G, Oturan MA. Removal of residual anti-inflammatory and analgesic pharmaceuticals from aqueous systems by electrochemical advanced oxidation processes. A review. Chemical Engineering Journal. 2013;228:944–64. doi: 10.1016/j.cej.2013.05.061

[6] Kanakaraju D, Glass BD, Goh PS. Advanced oxidation process-mediated removal of pharmaceuticals from water: a review of recent advances. Environ Sci Pollut Res Int. 2025;32(24):14316–50. doi: 10.1007/s11356-025-36547-5 40434594 PMC12202666

[7] Mahmoud WMM, Rastogi T, Kümmerer K. Application of titanium dioxide nanoparticles as a photocatalyst for the removal of micropollutants such as pharmaceuticals from water. Current Opinion in Green and Sustainable Chemistry. 2017;6:1–10. doi: 10.1016/j.cogsc.2017.04.001

[8] Zhang J, Fu D, Xu Y, Liu C. Optimization of parameters on photocatalytic degradation of chloramphenicol using TiO2 as photocatalyst by response surface methodology. J Environ Sci (China). 2010;22(8):1281–9. doi: 10.1016/s1001-0742(09)60251-5 21179970

[9] Zhang J, Zhu G, Liu W, Xi Y, Golosov DA, Zavadski SМ, et al. 3D core-shell WO3@α-Fe2O3 photoanode modified by ultrathin FeOOH layer for enhanced photoelectrochemical performances. Journal of Alloys and Compounds. 2020;834:154992. doi: 10.1016/j.jallcom.2020.154992

[10] Beheshti F, M. A. Tehrani R, Khadir A. Sulfamethoxazole removal by photocatalytic degradation utilizing TiO2 and WO3 nanoparticles as catalysts: analysis of various operational parameters. Int J Environ Sci Technol. 2019;16(12):7987–96. doi: 10.1007/s13762-019-02212-x

[11] Nguyen TT, Nam S-N, Son J, Oh J. Tungsten Trioxide (WO3)-assisted Photocatalytic Degradation of Amoxicillin by Simulated Solar Irradiation. Sci Rep. 2019;9(1):9349. doi: 10.1038/s41598-019-45644-8 31249354 PMC6597549

[12] Yang L, Chen Z, Ma T, Zhang S, Dai W, Xiao X, et al. Efficient electrochemical dehalogenation of florfenicol without discharging toxic intermediates via direct electron transfer over electrochromic WO3. Chemical Engineering Journal. 2021;412:127481. doi: 10.1016/j.cej.2020.127481

[13] Quyen VT, Kim J, Park P-M, Huong PT, Viet NM, Thang PQ. Enhanced the visible light photocatalytic decomposition of antibiotic pollutant in wastewater by using Cu doped WO3. Journal of Environmental Chemical Engineering. 2021;9(1):104737. doi: 10.1016/j.jece.2020.104737

[14] Zhu W, Liu J, Yu S, Zhou Y, Yan X. Ag loaded WO3 nanoplates for efficient photocatalytic degradation of sulfanilamide and their bactericidal effect under visible light irradiation. J Hazard Mater. 2016;318:407–16. doi: 10.1016/j.jhazmat.2016.06.066 27450332

[15] Voigt M, Savelsberg C, Jaeger M. Photodegradation of the antibiotic spiramycin studied by high-performance liquid chromatography-electrospray ionization-quadrupole time-of-flight mass spectrometry. Toxicological & Environmental Chemistry. 2017;99(4):624–40. doi: 10.1080/02772248.2017.1280039

[16] Mzimela N, Tichapondwa S, Chirwa E. Visible-light-activated photocatalytic degradation of rhodamine B using WO3 nanoparticles. RSC Adv. 2022;12(53):34652–9. doi: 10.1039/d2ra06124d 36545610 PMC9717418

[17] Cao H, Zhao X-L, Li H, Iqbal M, Zhang D, Zhang P-F, et al. Evaluation of bond strength degradation between FRP bars and normal concrete under seawater exposure: An explainable modeling approach. Structures. 2025;79:109525. doi: 10.1016/j.istruc.2025.109525

[18] Li K, Du H, Liu L, Yang H, Fang J, Li D. Research progress of machine learning in the field of photocatalysis applications. Journal of Industrial and Engineering Chemistry. 2025;151:146–66. doi: 10.1016/j.jiec.2025.04.039

[19] Tunala S, Zhai S, Wu F, Chen Y-H. Machine learning in photocatalysis: accelerating design, understanding, and environmental applications. Sci China Chem. 2025;68(8):3415–28. doi: 10.1007/s11426-024-2656-6

[20] Jaison A, Mohan A, Lee Y-C. Machine learning-enhanced photocatalysis for environmental sustainability: Integration and applications. Materials Science and Engineering: R: Reports. 2024;161:100880. doi: 10.1016/j.mser.2024.100880

[21] Ge L, Ke Y, Li X. Machine learning integrated photocatalysis: progress and challenges. Chem Commun (Camb). 2023;59(39):5795–806. doi: 10.1039/d3cc00989k 37093605

[22] Kazim H, Sabri M, Al-Othman A, Tawalbeh M. Functionalized conducting polymers in photocatalysis and opportunities for artificial intelligence applications. Nano-Structures & Nano-Objects. 2024;40:101371. doi: 10.1016/j.nanoso.2024.101371

[23] Ali MM, Hossen MdA, Abd Aziz A. Progress in prediction of photocatalytic CO2 reduction using machine learning approach: A mini review. Next Materials. 2025;8:100522. doi: 10.1016/j.nxmate.2025.100522

[24] Khan H, Rigamonti MG, Patience GS, Boffito DC. Spray dried TiO2/WO3 heterostructure for photocatalytic applications with residual activity in the dark. Applied Catalysis B: Environmental. 2018;226:311–23. doi: 10.1016/j.apcatb.2017.12.049

[25] Khan H, Usen N, Boffito DC. Spray-dried microporous Pt/TiO2 degrades 4-chlorophenol under UV and visible light. Journal of Environmental Chemical Engineering. 2019;7(4):103267. doi: 10.1016/j.jece.2019.103267

[26] Nasuhoglu D, Yargeau V, Berk D. Photo-removal of sulfamethoxazole (SMX) by photolytic and photocatalytic processes in a batch reactor under UV-C radiation (λmax=254 nm). J Hazard Mater. 2011;186(1):67–75. doi: 10.1016/j.jhazmat.2010.10.080 21167641

[27] Chen T, Guestrin C. Xgboost: A scalable tree boosting system. In: 2016.

[28] Javed MF, Shahab MZ, Asif U, Najeh T, Aslam F, Ali M, et al. Evaluation of machine learning models for predicting TiO2 photocatalytic degradation of air contaminants. Sci Rep. 2024;14(1):13688. doi: 10.1038/s41598-024-64486-7 38871797 PMC11176179

[29] Hou X, Lu H, Wang X, Yu Z, Chen C, Xu K, et al. Predicting photocatalytic degradation efficiency of rhodamine: A machine learning-based model for composite catalyst screening. Journal of Water Process Engineering. 2025;72:107512. doi: 10.1016/j.jwpe.2025.107512

[30] Iqbal M, Zhao X-L, Li H, Zhang D, Zhang P-F, Zhao X, et al. Evaluating hydrothermal degradation of GFRP composites under sustained loading using explainable machine learning. Journal of Ocean Engineering and Science. 2025;10(6):982–1001. doi: 10.1016/j.joes.2025.05.005

[31] Lundberg S, Lee SI. A unified approach to interpreting model predictions. 2017.

[32] Babanajad SK, Gandomi AH, Alavi AH. New prediction models for concrete ultimate strength under true-triaxial stress states: An evolutionary approach. Advances in Engineering Software. 2017;110:55–68. doi: 10.1016/j.advengsoft.2017.03.011

[33] Iqbal M, Zhang D, Jalal FE, Faisal Javed M. Computational AI prediction models for residual tensile strength of GFRP bars aged in the alkaline concrete environment. Ocean Engineering. 2021;232:109134. doi: 10.1016/j.oceaneng.2021.109134

[34] Sarafraz M, Amini MM, Adiban M, Eslami A. Facile synthesis of mesoporous black N–TiO2 photocatalyst for efficient charge separation and the visible-driven photocatalytic mechanism of ibuprofen degradation. Materials Science in Semiconductor Processing. 2020;120:105258. doi: 10.1016/j.mssp.2020.105258

[35] Toumpeki C, Vourekas A, Kalavrizioti D, Stamatopoulou V, Drainas D. Activation of bacterial ribonuclease P by macrolides. Biochemistry. 2008;47(13):4112–8. doi: 10.1021/bi701488q 18330998

[36] Ahmadpour N, Sayadi MH, Sobhani S, Hajiani M. Photocatalytic degradation of model pharmaceutical pollutant by novel magnetic TiO2@ZnFe2O4/Pd nanocomposite with enhanced photocatalytic activity and stability under solar light irradiation. J Environ Manage. 2020;271:110964. doi: 10.1016/j.jenvman.2020.110964 32778273

[37] Dou Y, Yan T, Zhang Z, Sun Q, Wang L, Li Y. Heterogeneous activation of peroxydisulfate by sulfur-doped g-C3N4 under visible-light irradiation: Implications for the degradation of spiramycin and an assessment of N-nitrosodimethylamine formation potential. J Hazard Mater. 2021;406:124328. doi: 10.1016/j.jhazmat.2020.124328 33144012

[38] Ounnar A, Favier L, Bouzaza A, Bentahar F, Trari M. Kinetic study of spiramycin removal from aqueous solution using heterogeneous photocatalysis. Kinet Catal. 2016;57(2):200–6. doi: 10.1134/s0023158416020087

[39] Krishnan S, Shriwastav A. Application of TiO2 nanoparticles sensitized with natural chlorophyll pigments as catalyst for visible light photocatalytic degradation of methylene blue. Journal of Environmental Chemical Engineering. 2021;9(1):104699. doi: 10.1016/j.jece.2020.104699

[40] Sohrabi MR, Ghavami M. Photocatalytic degradation of Direct Red 23 dye using UV/TiO2: Effect of operational parameters. J Hazard Mater. 2008;153(3):1235–9. doi: 10.1016/j.jhazmat.2007.09.114 18006230

[41] Sharifiyan MS, Fattah-alhosseini A, Karbasi M. Optimizing the hydrothermal post-treatment process for a TiO2/WO3 hybrid coating to enhance the photocatalytic degradation of methylene blue under visible light. Ceramics International. 2023;49(22):35175–85. doi: 10.1016/j.ceramint.2023.08.190

[42] Khan H, Swati IK. Fe3+-doped Anatase TiO2 with d–d Transition, Oxygen Vacancies and Ti3+ Centers: Synthesis, Characterization, UV–vis Photocatalytic and Mechanistic Studies. Ind Eng Chem Res. 2016;55(23):6619–33. doi: 10.1021/acs.iecr.6b01104

[43] Reinhard S, Rechberger F, Niederberger M. Commercially Available WO3 Nanopowders for Photoelectrochemical Water Splitting: Photocurrent versus Oxygen Evolution. Chempluschem. 2016;81(9):935–40. doi: 10.1002/cplu.201600241 31968792

[44] Bamwenda GR, Arakawa H. The visible light induced photocatalytic activity of tungsten trioxide powders. Applied Catalysis A: General. 2001;210(1–2):181–91. doi: 10.1016/s0926-860x(00)00796-1

[45] Farhadian M, Sangpour P, Hosseinzadeh G. Morphology dependent photocatalytic activity of WO3 nanostructures. Journal of Energy Chemistry. 2015;24(2):171–7. doi: 10.1016/s2095-4956(15)60297-2

[46] Fernandes SM, Barrocas BT, Nardeli JV, Montemor MF, Maçoas E, Oliveira MC, et al. Maximizing photocatalytic efficiency with minimal amount of gold: Solar-driven TiO2 photocatalysis supported by MICROSCAFS® for facile catalyst recovery. Journal of Environmental Chemical Engineering. 2024;12(2):112043. doi: 10.1016/j.jece.2024.112043

[47] Bekele T, Alamnie G. The photocatalytic degradation of organic pollutants-a comprehensive overview. Results in Chemistry. 2025;18:102758. doi: 10.1016/j.rechem.2025.102758

[48] Chekir N, Laoufi NA, Bentahar F. Spiramycin photocatalysis under artificial UV radiation and natural sunlight. Desalination and Water Treatment. 2014;52(34–36):6832–9. doi: 10.1080/19443994.2013.821632

[49] Vignati DA, Lofrano G, Libralato G, Guida M, Siciliano A, Carraturo F, et al. Photocatalytic ZnO-assisted degradation of spiramycin in urban wastewater: Degradation Kinetics and Toxicity. Water. 2021;:1051.

[50] Lofrano G, Libralato G, Casaburi A, Siciliano A, Iannece P, Guida M, et al. Municipal wastewater spiramycin removal by conventional treatments and heterogeneous photocatalysis. Sci Total Environ. 2018;624:461–9. doi: 10.1016/j.scitotenv.2017.12.145 29268218

